# Full-Length NAD^+^-I Riboswitches Bind a Single Cofactor but Cannot Discriminate against Adenosine Triphosphate

**DOI:** 10.1021/acs.biochem.3c00391

**Published:** 2023-11-10

**Authors:** Yoshita Srivastava, Maya E. Blau, Jermaine L. Jenkins, Joseph E. Wedekind

**Affiliations:** ‡ Department of Biochemistry & Biophysics and Center for RNA Biology, University of Rochester School of Medicine & Dentistry, Rochester, New York 14642, United States

## Abstract

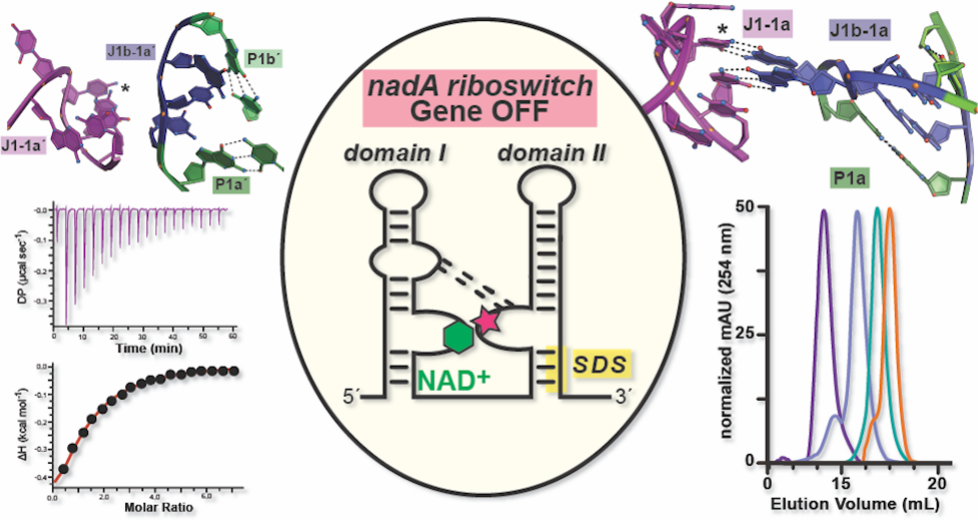

Bacterial riboswitches are structured RNAs that bind small metabolites to control downstream gene expression. Two riboswitch classes have been reported to sense nicotinamide adenine dinucleotide (NAD^+^), which plays a key redox role in cellular metabolism. The NAD^+^-I (class I) riboswitch stands out because it comprises two homologous, tandemly arranged domains. However, previous studies examined the isolated domains rather than the full-length riboswitch. Crystallography and ligand binding analyses led to the hypothesis that each domain senses NAD^+^ but with disparate equilibrium binding constants (*K*_D_) of 127 μM (domain I) and 3.4 mM (domain II). Here, we analyzed individual domains and the full-length riboswitch by isothermal titration calorimetry to quantify the cofactor affinity and specificity. Domain I senses NAD^+^ with a *K*_D_ of 24.6 ± 8.4 μM but with a reduced ligand-to-receptor stoichiometry, consistent with nonproductive domain self-association observed by gel-filtration chromatography; domain II revealed no detectable binding. By contrast, the full-length riboswitch binds a single NAD^+^ with a *K*_D_ of 31.5 ± 1.5 μM; dinucleotides NADH and AP_2_-ribavirin also bind with one-to-one stoichiometry. Unexpectedly, the full-length riboswitch also binds a single ATP equivalent (*K*_D_ = 11.0 ± 3.5 μM). The affinity trend of the full-length riboswitch is ADP = ATP > NAD^+^ = AP_2_-ribavirin > NADH. Although our results support riboswitch sensing of a single NAD^+^ at concentrations significantly below the intracellular levels of this cofactor, our findings do not support the level of specificity expected for a riboswitch that exclusively senses NAD^+^. Gene regulatory implications and future challenges are discussed.

## Introduction

**Figure 1:**
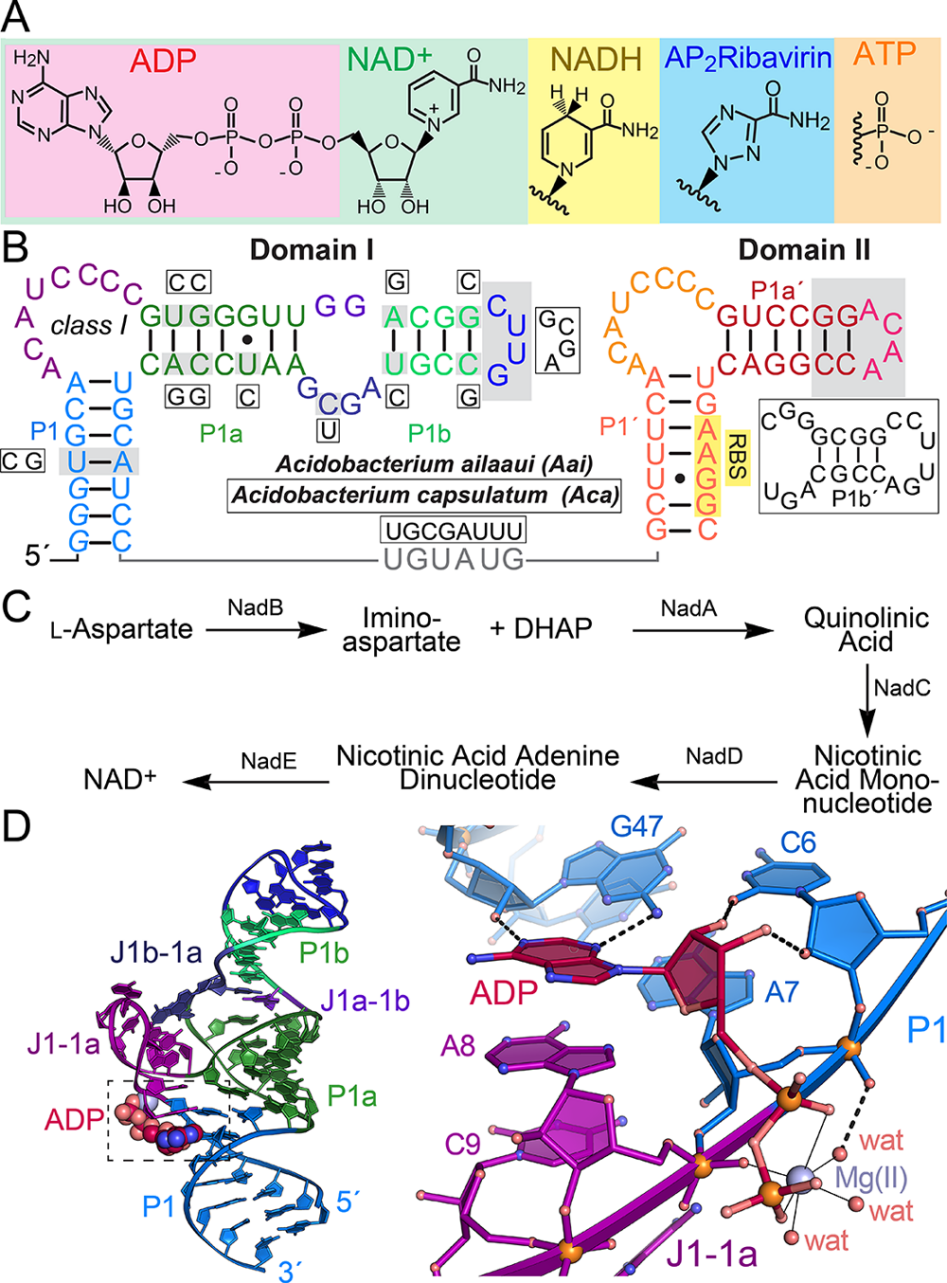
Schematic diagrams of NAD^+^ and related nucleotides, NAD^+^ riboswitches, *de novo* NAD^+^ biosynthesis, and molecular recognition of ADP. (a) Chemical structures of ADP (pink), NAD^+^ (green), NADH (yellow), AP_2_-ribavirin (cyan), and ATP (orange). The NADH (yellow) and AP_2_-ribavirin (cyan) substituents replace the nicotinamide ring of NAD^+^. For ATP, the γ-phosphate (orange) replaces the nicotinamide riboside moiety (green). (b) Secondary structure^9^ and full-length sequences of class I NAD^+^ riboswitches used in this investigation. Sequence differences between the *Aai* and *Aca* riboswitches are indicated by boxes shaded gray and with black borders. (c) The *de novo* NAD^+^ biosynthesis pathway requires genes *nadB*, *nadA*, and *nadC*. Gene products from *nadD* and *nadE* are indispensable for niacin salvage as well as *de novo* synthesis.^10^ (d) Ribbon diagram showing the overall domain I fold of the NAD^+^-I riboswitch with ADP, depicted as a space-filling model (PDB 6tf1).^11^ (Inset) Close-up view of the ADP binding pocket, showing the chemical basis of ADP recognition. The ADP moiety engages P1 using a type I A-minor interaction^12^ with stacking upon A8. A partially hydrated Mg(II) makes inner sphere contacts to the riboswitch backbone as well as additional water (wat)-mediated contacts. For clarity, the inset orientation is rotated ∼180° about the *x*-axis relative to the ribbon diagram (left).

Structured RNA motifs known as riboswitches play essential roles in small metabolite sensing, which is integral to the biochemical feedback circuitry of bacterial cells.^1−4^ Riboswitches have a bipartite organization that comprises an aptamer domain, which senses specific effectors, and an expression platform that changes conformation in response to effector binding, thereby controlling downstream gene expression.^5,6^ At present, >55 classes of riboswitches have been validated that bind numerous effector types, including nucleosides, sugars, amino acids, ions, and enzyme cofactors.^4,7,8^ In the latter category, NAD^+^ (Figure 1a) is a key effector. It is one of the most frequently used biochemical cofactors due to its participation in numerous metabolic reactions as well as redox sensing, signaling, and regulation.^13,14^ NAD^+^ functions in concert with its reduced form, NADH, which carries an electron-rich hydride moiety that participates in a myriad of reduction reactions (Figure 1a). In many bacteria, the NAD^+^/NADH redox pair is essential to feed electrons from reduced carbon metabolites into the electron transport chain that generates a proton gradient.^15,16^ Despite the prevalence and importance of NAD^+^ in biology, the identification of riboswitches that sense this pervasive cofactor remained relatively elusive.

Recently, the first known NAD^+^-sensing riboswitch was described (i.e., the *nadA* riboswitch or NAD^+^-I).^9,17^ The consensus secondary structure model for this riboswitch reveals two tandem stem-loop domains (Figure 1b). An intriguing aspect of the NAD^+^-I riboswitch is that each domain is believed to participate in the formation of a single binding pocket that senses a lone NAD^+^ or derivative thereof.^9^ The riboswitch was identified originally as a bulged stem-loop preceding *nadA* genes, which reside on the pathway for *de novo* NAD^+^ biosynthesis (Figure 1c). The *nadA* motif is always followed by a nearly identical bulged stem-loop, leading to the determination that the full-length *nadA* riboswitch comprises tandem, bulged stem-loop domains.^9^ This work further showed that repression of the downstream gene occurs when NAD^+^ is abundant and both tandem bulge-loop domains are present. Domain II of the riboswitch contains a ribosome binding site (RBS) in stem P1′ that is believed to control translation, making this a “gene off” regulator.^9^ Accordingly, the *nadA* motif appears to control translation of the downstream *nadA* gene encoding quinolinate synthase. The latter enzyme is conserved in all prokaryotes,^10^ where it converts iminoaspartate and dihydroxyacetone phosphate into quinolinic acid, which is an essential step in *de novo* NAD^+^ production (Figure 1c). Control of the *nadA* gene has broader implications for many metabolic processes that depend on the resulting dinucleotide cofactor. Indeed, hundreds of distinct enzymes bind NAD^+^ for individual biochemical reactions.^13^ NAD^+^ is also an essential cofactor in glycolytic substrate-level phosphorylation. It is a key component of ADP-ribosylation, a post-translational modification that controls numerous cellular processes^18^ as well as the underlying basis for toxicity by the diphtheria toxin and exotoxin A.^19^ The centrality of NAD^+^ in the core metabolism of all free-living organisms suggests the cofactor has ancient origins.^20^ The fact that so many riboswitches sense nucleotide-derived cofactors used by modern enzymes implies that ancient RNAs likely used these molecules, or derivatives thereof, in a prebiotic metabolism.^21^ As such, studying extant riboswitches that sense NAD^+^ provides insight into the structure and domain organization of existing gene-regulatory RNAs as well as bygone RNA molecules that utilized this pervasive cofactor in an RNA world.

To elucidate the mode of NAD^+^ binding and cofactor sensing by the NAD^+^-I riboswitch, two groups independently determined the cocrystal structures of domain I from related species. Each structure reveals the chemical determinants by which the riboswitch aptamer recognizes the adenosine diphosphate moiety of the cofactor, but neither structure reliably revealed the nicotinamide riboside moiety (Figure 1d).^11,22^ Prior biochemical studies suggested a role for domain II in sequestration of the expression platform, but in-line probing did not produce evidence of cofactor binding by domain II.^9^ Despite this, it was hypothesized that domain II interacts with the nicotinamide moiety of the dinucleotide. By contrast, domain I was shown to be essential in binding the ADP moiety of the cofactor.^9^ Structural analysis of domain I from *Candidatus koribacter versatilis* suggested that each isolated domain should be capable of recognizing its own individual NAD^+^ due to the high sequence identity of the respective bulge loops observed in the domain I complex with ADP.^11^ This hypothesis was supported further when cocrystal structures were determined for the individual domains of the NAD^+^-I riboswitch from *Acidobacterium capsulatum* in complex with ADP.^22^ This finding led to a proposed regulatory model in which the tandem domains each individually sense a single NAD^+^ but with disparate affinities of 127 μM and 3.4 mΜ.^22^ Accordingly, domain I was posited to bind cotranscriptionally and with tighter affinity at reduced cofactor concentrations, allowing continued gene expression. At high cofactor concentrations, domain II was proposed to bind NAD^+^, resulting in sequestration of the ribosome binding site (RBS) yielding a gene-off state.^22^ The latter gene regulatory model, however, established a dichotomy in the field. Specifically, the need to distinguish between one-NAD^+^-per-riboswitch^9^ versus two-NAD^+^-per-riboswitch binding modes.^22^ A second prediction is that the full-length riboswitch should discriminate against the γ-phosphate of ATP but favor NAD^+^ recognition because these nucleotides are present at concentrations of 0.12–9.6 mM and 1.6–2.6 mM in growing bacteria.^23,24^

Here, we build upon previous biochemical and structural analyses of the *nadA* motif with the goal of quantifying cofactor binding stoichiometry, affinity, and specificity for the full-length NAD^+^-I riboswitch, which has not been studied appreciably in previous investigations.^9,11,22^ Accordingly, we measured riboswitch interactions with NAD^+^, NADH, ADP, ATP, and the dinucleotide analogue AP_2_-ribavirin. This work was conducted using individual domain I and II constructs as well as the full-length riboswitch (Figure 1b). Our analysis revealed that domain I alone is competent to bind NAD^+^ and derivatives thereof, but domain II does not bind effectors even at elevated concentrations. Gel-filtration chromatography showed the propensity of individual domains to form monomer–dimer equilibria, which did not occur with the full-length riboswitch. Significantly, the full-length riboswitch binds a single NAD^+^ cofactor as well as ATP. Ligand binding appears localized entirely to domain I. Our observations support a one-NAD^+^-per-riboswitch model of cofactor recognition,^9^ in contrast to prior calls for a two-NAD^+^-per-riboswitch model that features a cryptic binding site in domain II.^22^ Moreover, our results show that the full-length NAD^+^-I riboswitch cannot discriminate against ATP. These findings are discussed in light of existing gene regulatory models.

## Experimental Methods

### *In Vitro* Transcription and Purification of the Full-Length *Aai* NAD^+^-I Riboswitch

A DNA template was designed as described^25^ using the *Aai* riboswitch sequence (Figure 1b). The natural tetraloops of each domain were replaced with GAAA tetraloops with CG closing pairs to promote stability.^26^ A PAGE-purified DNA ultramer (IDT, Inc.) template sequence was used: 5′-TAATACGACTCACTATA*GGC*TGCAACATCCCCGTGGGTTGGACGGCGAAAGCCGTAGCGAATCCACTGCAGCCTGTATGGCTTTCAACATCCCCGTCCGCGAAAGCGGACTGAAGGC, where italics indicate the T7 transcription start site and tetraloops are underlined. The sequence *GGC* was added in P1 to promote a strong transcription start,^27^ and tandem 2′-*O*-methyl groups were used at the 3′-end to reduce untemplated transcription.^28^ The lyophilized desalted template strand was dissolved in water at a concentration of 100 μM. A 17-mer PAGE-purified T7 DNA primer^27^ at 100 μM was annealed to the template in 1 mL to give 2 μM primer, 2 μM template, 10 μM MgCl_2_, and 0.20 M Na-HEPES at pH 7.0. The reaction tube was mixed gently by inversion, followed by heating at 95 °C for 3 min prior to slow cooling to room temperature. A 10 mL transcription was performed in a reaction comprising 0.075 M Na-HEPES at pH 7.5, 0.067 M DTT, 0.03 M MgCl_2_, 21% (w/v) PEG 3350, 0.002 M spermidine-HCl, 0.0063 M ATP, 0.0063 M UTP, 0.0063 M CTP, 0.008 M GTP, 1 mL of annealed template, 0.26 mg mL^–1^ BSA, and 0.027 mg mL^–1^ T7 polymerase, as described.^25^ The reaction tube was inverted gently again to achieve mixing and incubated for 5 h at 37 °C. The reaction was spun at 10 000 RCF for 10 min at room temperature. The supernatant was ethanol-precipitated and stored at −20 °C. The RNA was purified by batch chromatography using Toyoperl DEAE-650 M resin (Sigma-Aldrich, Inc.) followed by separation using denaturing 6% PAGE. RNA bands were visualized by UV with exposure precautions,^29^ excised, and eluted.^25^ A second DEAE-650 M column was used to remove contaminants followed by desalting on a PD-10 column (GE Healthcare). Purity was assessed by analytical PAGE stained by SYBR Gold (Thermo-Fisher) and visualized on a Gel Doc XR+ instrument (BioRad). The estimated purity was >99%. The yield per 10 mL of synthesis was 3 mg of RNA based on spectrophotometry at 260 nm. The RNA was stored as a lyophilized powder at −20 °C.

### Purification of Individual *Aca* Class I Domains

Each class I domain was generated by chemical synthesis (Horizon Discovery PLC, Lafayette, CO). We designed two constructs for domain I. A single-stranded domain I construct was synthesized as a 54-mer. The RNA sequence was 5′-GGCGGCAACAUCCCCGCCGGUUGGGCGCGAAAGCGCAGUGAACCGGCUGCAGCC. A 50-mer split domain I construct was also produced from two RNA strands comprising the wildtype *Aca* sequence; the construct comprises a 28-mer strand with the sequence 5′-GGGCGCAACAUCCCCGCCGGUUGGGCGC and a 22-mer with the sequence 5′- GCGCAGUGAACCGGCUGCGUCC. The sequence of P1 was modified in a nonconserved segment to promote duplex formation. A single-strand domain II 50-mer hairpin was synthesized similarly and had the sequence 5′-GCUUUCAACAUCCCCGUCCCGGGCGGCCUUGACCGCAGUGGACUGAAGGC (Figure 1b). A split 48-mer construct of domain II was prepared that comprises 28-mer and 20-mer sequences. The 28-mer sequence was 5′-GCUUUCAACAUCCCCGUCCCGGGCGGAG and the 20-mer was 5′-CUCCGCAGUGGACUGAAGGC. Deprotection of each strand was performed as described by the manufacturer except that heating was for 1 h at 65 °C. Each strand was purified by 20% denaturing PAGE followed by DEAE-650 M chromatography.^25^ After ethanol precipitation of the pooled DEAE fractions, the RNA was dissolved in water and desalted on a PD-10 column (GE Healthcare). Each RNA strand was stored as a lyophilized powder at −20 °C.

### Analysis of RNA Secondary Structure

The free energy associated with domain I and domain II folding as monomers and intermolecular dimers was evaluated using the “predict secondary structure” and “bimolecular” options of RNAstructure (v6.0.1).^30,31^ For the bimolecular structures, we used two folding algorithms. The Duplex fold option was chosen because it predicts the lowest energy structure for two interacting sequences while not allowing intramolecular WC base pairing. The Bifold algorithm predicts the lowest energy structure for two interacting sequences, allowing for intramolecular WC base pairing. In each case, the default options were selected for folding. The secondary structures are depicted in Figure S1. The propensity of individual strands to adopt hairpins was comparatively weak relative to the bimolecular split constructs that form from the 28-mer and 22-mer strands that compose domain I or the 28-mer and 20-mer strands that compose domain II. The corresponding free energies are provided in the legend of Figure S1.

### Mass Spectrometry

To confirm the presence of each ligand from commercial sources, each effector was subjected to mass spectrometry using a TSQ Quantis triple quadrupole system (Thermo Scientific) at the SUNY Upstate Medical Center (Syracuse, NY) or a Q Exactive Plus Hybrid Quadrupole-Orbitrap instrument (Thermo Scientific) at the University of Rochester (Rochester, NY). Exact masses were from the manufacturers. The ADP sample (Selleck Chemicals LLC) was analyzed in negative ion mode. The exact mass is 427.03 Da for the negative ion. The species was observed at an *m*/*z* value of 426.01 (Figure S2a). The spectrum indicated several non-nucleotide, singly charged negative ion contaminants.^32^ NAD^+^ (MedChem Express LLC) was analyzed in positive ion mode.^32^ The exact mass of the protonated species is 664.12 Da, in agreement with the observed value at an *m*/*z* value of 664.11 (Figure S2b). The exact mass of NADH (MedChem Express LLC) is 665.13 Da for the negatively charged species. The sample was analyzed in negative ion mode. The observed major species was doubly charged at an *m*/*z* value of 663/2 = 331.5 (Figure S2c). The exact mass of AP_2_-ribavirin (Jena Bioscience) is 653.10 Da for the negatively charged species. The sample was analyzed in negative ion mode. The observed major species was doubly charged at an *m*/*z* value of 651/2 = 325.5 (Figure S 2d). The ATP sample (MedChem Express LLC) was analyzed in positive ion mode. The exact mass is 507.00 for the neutral species, in agreement with the major species observed at an *m*/*z* of 508.00 (Figure S2e).

### Isothermal Titration Calorimetry

**Figure 2:**
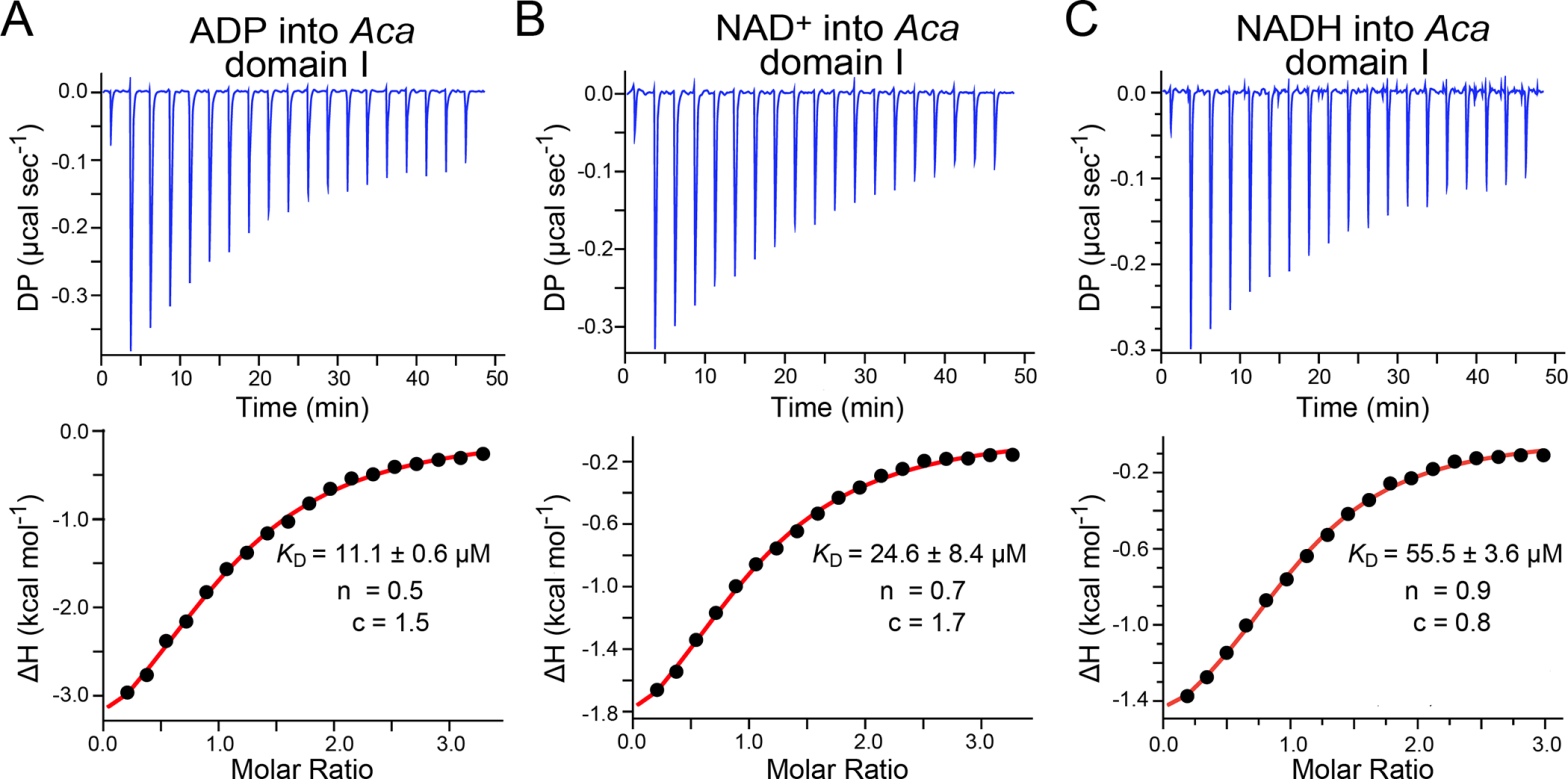
Representative ITC thermograms and curve fits for effector binding to *nadA* domain I *Aca* split constructs. (a) ADP binding. (b) NAD^+^ binding. (c) NADH binding. Here and elsewhere, the individual *K*_D_, *n*, and *c* values are shown for each run. Average thermodynamic parameters from replicate titrations are given in Table 1.

**Figure 3:**
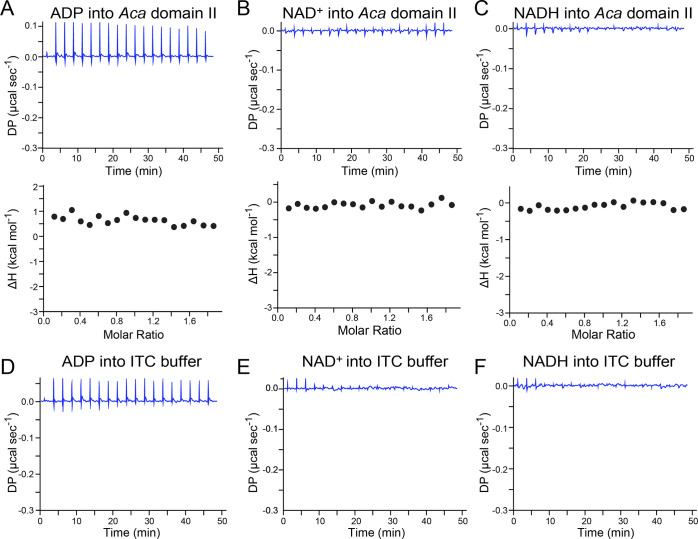
Representative ITC thermograms for effector titration into *Aca* split domain II or buffer. (a) ADP titrated into domain II. (b) NAD^+^ titrated into domain II. (c) NADH titrated into domain II. (d–f) Various effectors were titrated into ITC buffer containing no riboswitch.

**Figure 4:**
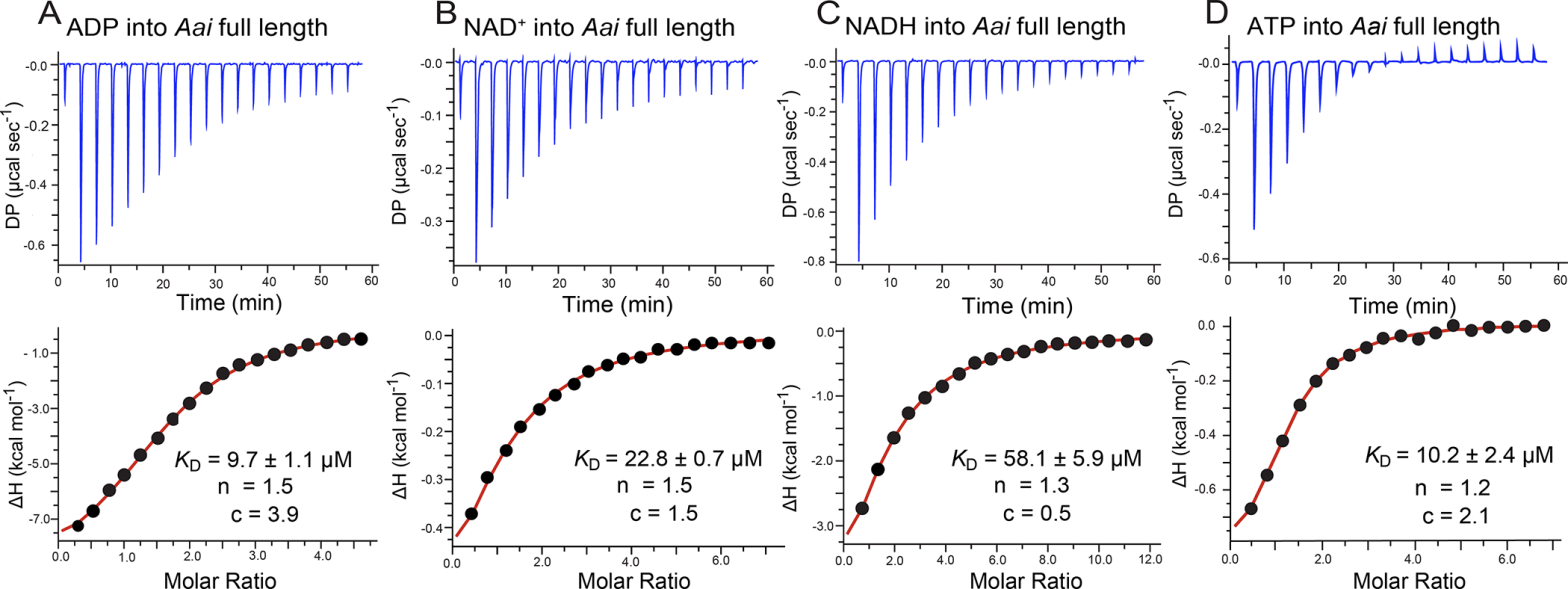
Representative ITC thermograms and curve fits for effector binding to the full-length *Aai* riboswitch. (a) ADP binding. (b) NAD^+^ binding. (c) NADH binding. (d) ATP binding. Average thermodynamic parameters from replicate titrations are given in Table 1.

Titrations were conducted using a PEAQ-ITC instrument (Malvern Panalytical, UK) with samples held at 10 °C and an injection spacing of 180 s. The technical injection was 0.4 μL, and the ensuing injections were 2 μL each. Lyophilized strands of the split *Aca* domain I construct were each dissolved in 75 μL of 0.01 M Na-cacodylate at pH 7.0. The concentration of each dissolved domain I or domain II sample was determined spectrophotometrically at 260 nm based on the extinction coefficients supplied by Horizon Discovery (ε_260_ = 252.5 mM^–1^ cm^–1^ for the domain I 28-mer, ε_260_ = 201.6 mM^–1^ cm^–1^ for the domain I 22-mer, ε_260_ = 257.5 mM^–1^ cm^–1^ for the domain II 28-mer, and ε_260_ = 190.0 mM^–1^ cm^–1^ for the domain II 20-mer). An ε_260_ of 1129.4 mM^–1^ cm^–1^ was used for the full-length 100-mer based on the OligoSpec Calculator (https://www.biosearchtech.com/oligospec-calculator-6628), which corrects the ε_260_ for hypochromicity. For the split constructs of domains I and II, the more concentrated strand was diluted using 0.01 M Na-cacodylate at pH 7.0 to match the concentration of the more dilute strand. A volume of 75 μL of each strand was combined to produce an equimolar solution that was heated to 65 °C for 3 min. Lyophilized strands of the *Aca* domain II and the full-length *Aai* NAD^+^-I riboswitch were dissolved similarly in 75 μL of 0.01 M Na-cacodylate at pH 7.0 and heated to 65 °C for 3 min. An equal volume of preheated folding buffer (0.01 M Na-cacodylate at pH 7.0, 0.1 M NaCl, and 0.04 M MgCl_2_) was added slowly to the heated RNA, and the combined solution was heated for an additional 3 min at 65 °C followed by slow cooling to 24 °C. Each equilibrated sample was subjected to overnight dialysis at 4 °C against 2 L of ITC buffer comprising 0.10 M Na-HEPES at pH 7.0, 0.10 M NaCl, and 0.02 M MgCl_2_ using a 3500 MWCO Slide-A-Lyzer Cassette G2 (Thermo-Fisher Scientific). Each effector was weighed on an analytical balance and dissolved in the used ITC buffer. The final concentration was verified spectrophotometrically by using known ligand extinction coefficients at 260 nm. The ligand in the syringe was kept in a concentration range of 0.47–1.05 mM. The domain I sample in the cell was kept in a concentration range of 0.030–0.060 mM. For the *Aca* domain II and *Aai* full-length samples, the ligand in the syringe was kept in a concentration range of 0.40–0.63 mM and the RNA in the sample cell was kept at a concentration of 0.025–0.040 mM. All thermograms were analyzed with MicroCal PEAQ-ITC analysis software (Malvern Panalytical) using a 1:1 binding model. This model was chosen because it produced the best thermogram fits in terms of the visual match of the data to the isotherm and the χ^2^ values indicating goodness of fit, as described.^33^ Binding simulations were performed using MicroCal PEAQITC analysis software or SEDPHAT^34^ (Figure S3). Values of Δ*H* were obtained directly from thermograms. A fit of each thermogram through the inflection point yielded the apparent equilibrium association constant, *K*_A_ (where *K*_A_ = *K*_D_^–1^). Using the equation Δ*G* = Δ*H* – *T*Δ*S*, the Gibbs free energy was calculated along with the entropy term, −*T*Δ*S*, where *T* is the temperature.^35^ The sample concentration and *n* value (i.e., ligand-to-receptor ratio) were allowed to vary during the final fitting process. Given the restraints available from the known concentration of ligand and receptor, the use of the full thermogram range for fitting, an adequate signal-to-noise ratio from heats of titration, and the application of specific binding models, the thermograms could be reliably fit despite low *c* values^36^ that were in a range from 0.6–3.1. Duplicate or greater replicates of each ITC run were performed for each effector. Titrations of effector into buffer were performed as controls for heats of dilution (Figure S4). These control titrations were subtracted as the mean or point-by-point corrections from the heats of injection for each thermogram, although this did not yield appreciably different background corrections compared with the fitted offset corrections calculated by MicroCal PEAQITC analysis software, which was the method chosen for analysis herein. Representative thermograms are provided in Figures 2, 3, and 4, and the average results from ≥2 technical replicates are in Table 1.

### Size Exclusion Chromatography

RNA constructs were evaluated for multimerization by size-exclusion chromatography. A Superose 6 10/300GL column (Cytiva Life Sciences) was standardized by using in-house RNAs with known molecular weights and oligomeric states. The standards included HIV-1 TAR 27-mer (MW 8208 Da),^37^ the *Kpn* preQ_1_-I_III_ riboswitch 46-mer (MW 14017 Da), the *Lrh* preQ_1_-II riboswitch 77-mer (MW 23442 Da),^38^ and the dimeric *Fpr* preQ_1_-III riboswitch 101-mer (MW 61693.0 Da).^39^ Each NAD^+^-I RNA sample was dissolved in 0.05 M Na-HEPES at pH 7.0 and heated at 65 °C, followed by the addition of an equal volume of preheated folding buffer comprising 0.05 M Na-HEPES at pH 7.0, 0.1 M NaCl, 0.04 M MgCl_2_, and 0.24 M NAD^+^. Each sample was warmed for an additional 3 min at 65 °C and then slow cooled in a heating block to 24 °C. For size exclusion, the column was equilibrated with a running buffer of 0.05 M Na-HEPES at pH 7.0, 0.1 M NaCl, 0.02 MgCl_2_, and 0.24 M NAD^+^. A sample volume of 100 μL was injected manually into the column. SEC was conducted on hairpin sequences of domains I and II (Figure S5). Due to the propensity of each hairpin domain sequence to fold as a monomer–dimer equilibrium (noted above and in Figures S1a, b, e, and f), these samples were excluded from the ITC analysis. A control sample of the full-length *Aai* riboswitch without a ligand was run in the unbound state under identical buffer conditions (Figure S6). Each sample was eluted in running buffer as 1 mL fractions and monitored by in-line absorption at 254 and 280 nm.

### Domain Interface Analysis

We used the program PISA^40^ to identify interfaces and contacts in domains I and II based on existing crystal structures retrieved from the PDB (Figure S7). The coordinates analyzed were 6tf1 (domain I with ADP bound), 7d81 (domain II with NAD^+^), 7d7v (U1A in complex with domain I with NAD^+^), and 7d7w (domain I with NAD^+^). Although multiple interfaces were identified, we focused on those involving junctions because these regions are conserved, are important for ligand binding, are functionally relevant, and have the potential to provide insight into interdomain pairing. Nucleotides and ions were included in the calculations, but cofactors were not included due to their negligible contribution in the cases described.

## Results

### Cofactors Bind to Domain I with High Affinity

Previous work described the discovery and validation of the *nadA* motif, which suggested that the riboswitch senses NAD^+^ inside cells to regulate the *nadA* gene encoding quinolinate synthase A (Figure 1c).^9^ To investigate *nadA* riboswitch affinity and specificity, we started with an analysis of ligand binding to domain I. Due to the potential for misfolding through stable intermolecular WC base pairing between domain I hairpin sequences (Figures S1a–c), we chose to use a “split construct” of domain I that comprises a 28-mer paired through WC interactions with a 22-mer, giving rise to a molecule devoid of a joining hairpin loop (Figure S1d). Importantly, split constructs avoid the well-known problem of forming self-complementary duplexes from hairpin sequences due to intermolecular WC pairing.^41−43^ The split domain I construct is also more stable energetically than the intramolecular hairpins formed by individual 28-mer and 22-mer strands (Figure S1d). Importantly, the hairpin loop that was removed from the split domain I construct is not conserved in the consensus model^9^ and does not participate in cofactor binding in known crystal structures.^11,22^

Previous structural analyses of domain I revealed that the adenosine diphosphate moiety of NAD^+^ interacts with domain I at the P1/J1-1a transition point^11,22^ (Figure 1d). The adenine of NAD^+^ stacks upon A7 and A8, slightly resembling an adenosine platform comprising A225 and A226 of the *Tetrahymena* group I intron P4–P6 domain.^44^ However, recognition of the ligand’s nucleobase occurs via a type I A-minor motif wherein the adenine hydrogen bonds to the G47 sugar edge of domain I while its ribose hydrogen bonds to the sugar edge of C6. The adenine of the ligand binds the minor groove in an inclined manner with respect to the plane of the C6-G47 base pair, as observed in other type I A-minor motifs.^38,39^ Notably, the available cocrystal structures do not reveal the mode of binding by the nicotinamide riboside of NAD^+^. This region is disordered in electron density maps,^11,22^ which agrees with in-line probing data on domain I, in which ADP showed indistinguishable scission patterns compared to NAD^+^.^9^

For the analysis here, we chose ITC because it is a label-free approach that allows for direct measurement of Δ*H* for a given receptor–ligand interaction.^35,45^ For parity with previous studies, we chose to investigate riboswitch sequences from *Acidobacterium capsulatum* (*Aca*) and *Acidobacterium ailaaui* (*Aai*), which were analyzed for ligand binding in previous independent investigations.^9,22^ The *Aca* and *Aai* sequences exhibit all of the conserved nucleotides and secondary structure features present in the consensus model.^9^ First, we tested the binding of *Aca* domain I to three related ligands: ADP, NAD^+^, and NADH (Figure 1a). Our rationale for this was that each ligand contains an adenosine diphosphate moiety that interacts with domain I, whereas NAD^+^ and NADH each contain a nicotinamide riboside moiety (Figure 1a), predicted to be recognized by domain II.^9^ The results revealed an average *K*_D_ value of 11.1 ± 0.6 μM for ADP and a 0.5:1 ligand-to-receptor ratio (Figure 2a and Table 1). Binding indicated a favorable enthalpic component (Δ*H* of −11.8 ± 1.8 kcal mol^–1^) but an unfavorable entropic component (−*Τ*Δ*S* of +5.3 ± 1.8 kcal mol^–1^). Interestingly, NAD^+^ and NADH showed average *K*_D_ values of 24.6 ± 8.4 and 55.5 ± 3.6 μM (Figures 2b and c and Table 1). NAD^+^ produced a favorable enthalpic contribution of −9.0 ± 4.7 kcal mol^–1^ but an unfavorable entropy of +3.0 ± 5.0; by contrast, the NADH enthalpy (−4.3 ± 0.6 kcal mol^–1^) and entropy (−1.3 ± 0.6 kcal mol^–1^) contributions were each favorable (Table 1). Notably, the ligand-to-receptor ratios of 0.7 and 0.9 for NAD^+^ and NADH were higher than those for ADP (Table 1). In each case, the presence of the nicotinamide riboside resulted in poorer binding to domain I compared to ADP (Figure 2a and Table 1). Such a result is unexpected if the nicotinamide riboside moiety is not recognized by the ligand binding pocket, as suggested by the domain I cocrystal structures (Figure 1d). Accordingly, this observation and the substoichiometric ligand-to-receptor ratios suggest that individual domain I molecules self-interact (discussed below). Otherwise, the thermodynamic properties resemble metabolite binding by other riboswitches, such as the preQ_1_-II riboswitch.^46^

**Table 1:** Average NAD^+^-I Riboswitch Thermodynamic Parameters

sample	ligand	*K*_D_ (μM)	*n*	Δ*H* (kcal mol^–1^)	–TΔ*S* (kcal mol^–1^)	Δ*G* (kcal mol^–1^)	*c*	*K*_rel_^a^
dom I	ADP	11.1 ± 0.6^b^	0.5 ± 0.01	–11.8 ± 1.8	5.3 ± 1.8	–6.4 ± 0.01	1.5	0.4
dom I	NAD^+^	24.6 ± 8.4	0.7 ± 0.01	–9.0 ± 4.7	3.0 ± 5.0	–6.0 ± 0.20	1.7	0.8
dom I	NADH	55.5 ± 3.6	0.9 ± 0.10	–4.3 ± 0.6	–1.3 ± 0.6	–5.5 ± 0.00	0.8	1.8
dom I	AP_2_- ribavirin	21.9 ± 8.4	0.6 ± 0.10	–7.7 ± 1.4	1.6 ± 1.6	–6.1 ± 0.20	1.1	0.7
full-length	ADP	9.7 ± 1.1	1.5 ± 0.01	–9.9 ± 0.6	3.4 ± 0.7	–6.5 ± 0.10	3.3	0.3
full-length	NAD^+^	31.5 ± 1.5	1.0 ± 0.01	–8.4 ± 0.01	2.4 ± 0.01	–6.0 ± 0.02	0.8	N/A
full-length	NADH	59.7 ± 2.2	1.4 ± 0.01	–7.1 ± 0.9	1.7 ± 0.9	–5.5 ± 0.02	0.5	1.9
full-length	AP_2_-ribavirin	30.2 ± 1.2	1.1 ± 0.10	–9.1 ± 0.7	3.2 ± 0.8	–5.9 ± 0.02	1.0	1.0
full-length	ATP	11.0 ± 3.5	1.2 ± 0.11	–10.9 ± 0.9	4.4 ± 1.1	–6.4 ± 0.18	1.0	0.4
dom II	ADP	no binding	n/d^c^	n/d	n/d	n/d	n/d	n/d
dom II	NAD^+^	no binding	n/d	n/d	n/d	n/d	n/d	n/d
dom II	NADH	no binding	n/d	n/d	n/d	n/d	n/d	n/d

a *K*_rel_ is the ratio of the apparent equilibrium binding constant of a ligand variant to NAD^+^ binding by the wildtype full-length NAD^+^-I riboswitch.

b Errors are the standard error of the mean. All measurements were performed two or more times.

c n/d means not detectable.

### Cofactors Bind More Tightly to Domain I Compared to Previous Experiments

The equilibrium binding of ADP to domain I measured here, with a *K*_D_ of 11.1 ± 0.6 μM (Table 1), was ∼8-fold stronger than previous values measured by ITC or fluorescence, which were 95 and 94 μM, respectively.^22^ The latter measurements were conducted on domain I of the *Aca* riboswitch, which is used herein. Similarly, an independent study on domain I of the *Ckv* riboswitch reported a value of 89 μM from ITC.^11^ Notably, the value of ∼60 μM from in-line probing^9^ of domain I from *Eag* is closer to our observations. Similarly, the *K*_D_ of 24.6 ± 8.4 μM measured here for *Aca* domain I binding to NAD^+^ was stronger than previous independent studies that reported *K*_D_ values of 127 μM^22^ for *Aca* domain I and >150 μM^11^ for *Ckv* domain I by ITC.

As in other studies, we measured a lower affinity for NADH (*K*_D_ of 55.5 ± 3.6 μM) compared to that for related ligands. An independent analysis of the *Aca nadA* domain I by ITC revealed a *K*_D_ of 305 μM.^22^ Similarly, in-line probing of domain I from *Aai* and *Eag* yielded *K*_D_ values of ∼300 and ∼200 μM.^9^ Although our analysis corroborates the observation that NADH binds most poorly to domain I among the nucleotides analyzed herein, our results showed ∼3.6–5.5-fold better binding based on our average *K*_D_ of 55.5 ± 3.6 μM (Table 1). Despite a high level of sequence conservation among all domain I constructs in each of these studies, two additional differences must be considered. First, we recorded all ITC measurements at 10 °C to improve the stability of the riboswitch constructs. We hypothesized that tighter binding would lead to higher quality thermograms that allow more accurate fits, which are needed to obtain reliable *K*_D_ values.^36^ By contrast, other studies were conducted at higher temperatures in a range of 20–25 °C.^9,11,22^ Second, previous binding experiments utilized different pH values (e.g., 8.3 for in-line probing and 7.0 for ITC), monovalent salts (e.g., NaCl or KCl between 50 and 100 mM), and different Mg^2+^ concentrations (between 10–20 mM). Nonetheless, our results support a binding model wherein domain I recognizes a single ADP moiety in the absence of domain II. This result agrees with published cocrystal structures (Figure 1d)^11,22^ as well as in-line probing results that showed indiscernible domain I cleavage patterns in the presence of ADP, NAD^+^, and NADH.^9^ Importantly, all binding studies of domain I support the affinity trend ADP > NAD^+^ > NADH regardless of variations in the sequence and binding conditions.

### Nucleotide Cofactors Do Not Bind Detectably to *Aca* Domain II

We next asked whether domain II of the *nadA* regulatory motif binds effectors. Our rationale was that previous analyses of domain II yielded mixed results in terms of nucleotide and cofactor binding. In-line probing is highly sensitive but showed no apparent modulation by NAD^+^.^9^ By contrast, independent fluorescence analyses of domain II suggested that ADP binds with a *K*_D_ of 3.4 mM.^22^ Accordingly, we analyzed *Aca* domain II binding to ADP, NAD^+^, and NADH. This bulge-loop domain is located downstream of domain I and exhibits a very similar sequence (Figure 1b). Like domain I, we used a split domain II construct to eliminate misfolded dimers caused by intermolecular WC base pairing (Figures S1e–h).

Importantly, we saw no appreciable heats of binding when ADP was titrated into domain II (Figure 3a). Similar results were obtained for NAD^+^ (Figure 3b) and NADH (Figure 3c). As a control, we injected each effector into the ITC buffer and observed heats of dilution comparable to the levels observed in the presence of domain II (Figures 3d–f). These experiments concur with prior observations from in-line probing that could not detect domain II modulation from NAD^+^ or analogues thereof.^9^ The results are also consistent with the observation that binding to domain II by ADP is very weak.^22^ Simulations suggested that a *K*_D_ of 1.0 mM would be detectable with our experimental apparatus, albeit incapable of being reliably fit, whereas a *K*_D_ of 3.4 mM would not be readily detected due to a limited heat of binding (Figures S3a and b). Accordingly, ADP binding affinity for domain II must be poorer than 1.0 mM, as reported,^22^ and is likely worse for NAD^+^ if domain II follows the trend observed for domain I wherein ADP binds more tightly than NAD^+^ or NADH (Table 1).

### The Full-Length Riboswitch Binds a Single Cofactor but Does Not Discriminate against ATP

We next considered the interaction of NAD^+^ with the full-length *Aai nadA* riboswitch. Our rationale was that ITC can provide an accurate ligand-to-receptor ratio^35^ and that the full-length riboswitch was not investigated in prior ITC or fluorescence experiments.^11,22^ We chose to examine the *Aai* riboswitch because it has a shorter 6 nt interdomain linker compared to the *Aca* riboswitch and domain II is devoid of a P1b′ helix (Figure 1b). Accordingly, the *Aai* riboswitch is shorter compared to several other full-length *nadA* motifs but maintains all conserved features identified in the consensus model.^9^

We first measured the affinity of the full-length *Aai* riboswitch for ADP. The results revealed an average *K*_D_ of 9.7 ± 1.1 μM, driven by favorable enthalpic (−9.9 ± 0.6 kcal mol^–1^) but unfavorable entropic (+3.4 ± 0.7 kcal mol^–1^) contributions (Figure 4a and Table 1). The affinity was comparable to *Aca* domain I alone (i.e., 11.1 ± 0.6 μM, Table 1), suggesting that the covalent attachment of domain II did little to enhance the affinity for the adenosyl moiety of the ligand. We next measured the binding of NAD^+^, which yielded a *K*_D_ of 31.5 ± 1.5 μM. Again, the binding was enthalpically driven (−8.4 ± 0.01 kcal mol^–1^) with an unfavorable entropy (+2.4 ± 0.01 kcal mol^–1^). Binding of the full-length *Aai* riboswitch to NAD^+^ was comparable to that of *Aca* domain I when the associated error was considered (i.e., 24.6 ± 8.4 μM, Table 1). NADH binding to the full-length *Aai* riboswitch yielded a *K*_D_ of 59.7 ± 2.2 μM, which agreed well with that of *Aca* domain I (*K*_D_ of 55.5 ± 3.6 μM, Table 1). Importantly, the ligand-to-receptor ratios for these experiments were found to be in a range of 1.0–1.5 (discussed below).

We next tested the binding of ATP to the full-length *Aai* riboswitch, which has not been tested previously. We hypothesized that the full-length riboswitch would discriminate against the γ-phosphate group of ATP as a basis to select NAD^+^ over ATP (Figure 1a). Surprisingly, the results revealed a *K*_D_ of 11.0 ± 3.5 μM with a ligand-to-receptor ratio of 1.2 (Figure 4d and Table 1). The thermodynamic profile is similar to that of other ligands. The finding that ATP binds to the full-length *Aai* riboswitch with slightly higher affinity than NAD^+^ (*K*_D_ of 31.5 ± 1.5 μM) prompted us to test the importance of K^+^ ions in our binding buffer, which yielded an ∼10-fold increase in effector affinity by the lysine riboswitch.^47,48^ However, we saw no significant change in NAD^+^ affinity when Na^+^ was replaced by K^+^, and the ligand-to-receptor ratio remained between 1.3 and 1.5 (Figure S8). The results suggest that the full-length *Aai* riboswitch does not discriminate between NAD^+^ and ATP under the conditions tested here. Moreover, monovalent ions such as Na^+^ and K^+^ do not affect the binding affinity for NAD^+^ through site-bound contacts mediated by the RNA and ligand. At present, it is unknown whether monovalent ions exclude ATP from binding.

### ITC Simulations of the Full-Length Riboswitch Support 1:1 Binding Stoichiometry

Given that our results overwhelmingly support a ligand-to-receptor ratio of unity for the full-length *Aai* riboswitch, we conducted ITC simulations to ascertain the feasibility of detecting two-site binding based on the experimental setup used herein. In one scenario, we assumed two high-affinity effector-binding sites with a *K*_D_1__ of 30 μM and a *K*_D_2__ of 50 μM, as reported,^22^ which produced a sigmoidal isotherm that does not saturate (Figure S3c). In a second scenario, we simulated one high-affinity site (*K*_D_1__ of 20 μM) and one low-affinity site, in agreement with both our data and previous low-affinity recognition (*K*_D_2__ of 3.4 mM) by domain II.^22^ This simulation shows early binding and produces a longer hyperbolic phase that does not saturate (Figure S3d). Based on visual inspection and the much poorer χ^2^ values of 275 (two high-affinity sites model) and 0.024 (one high-affinity and one low-affinity site) relative to the one-site model used in our analysis (χ^2^ of 0.0009), neither two-site model adequately described the observed experimental data. Importantly, both simulations produced isotherms that are distinct from those observed experimentally here (Figure 4a–d and filled circles in Figures S3c and d), consistent with a model in which one effector binds per full-length riboswitch.

### AP_2_-Ribavirin Binds to the Full-Length Riboswitch as Tightly as NAD^+^

Several analogues of ADP and a limited number of NAD^+^ mimics were analyzed for their binding affinity to the *nadA* riboswitch to identify functional groups important for cofactor recognition.^9^ To complement existing studies, we identified AP_2_-ribavirin as a ligand of interest to probe cofactor specificity because it was not explored previously. This bipartite molecule preserves the ADP moiety but replaces the nicotinamide group with a planar triazole ring attached to a pendant amide (Figure 1a), proffered as a key determinant of binding.^9^ Accordingly, AP_2_-ribavirin is a mimic of NAD^+^ but with a smaller, planar ring substitution at the site of hydride transfer.^49^ In contrast to that of NAD^+^, the triazole ring of AP_2_-ribavirin is uncharged.

**Figure 5:**
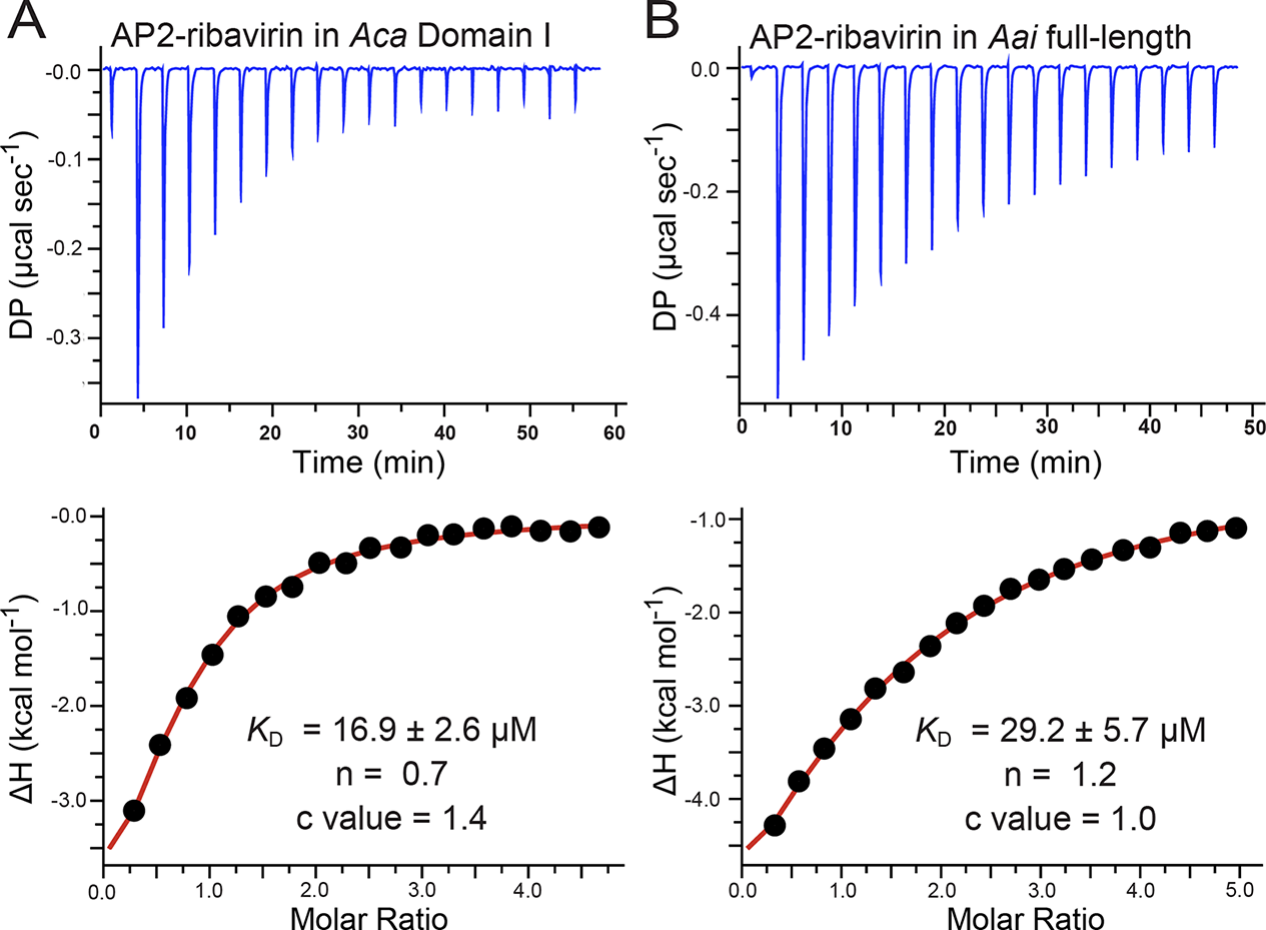
Representative ITC thermograms and curve fits for the binding of AP_2_-ribavirin to *Aca* domain I and *Aai* full-length. (a) Binding to the *Aca* riboswitch split domain I. (b) Binding to the *Aai* NAD^+^-I full-length riboswitch. Average thermodynamic parameters from replicate titrations are listed in Table 1.

Titration of AP_2_-ribavirin into *Aca* domain I produced a *K*_D_ of 21.9 ± 7.1 μM (Figure 5a and Table 1), which is nearly identical to NAD^+^ binding (*K*_D_ of 24.6 ± 8.4 μM). Binding was driven by a favorable enthalpy (Δ*H* of −7.7 ± 1.4 kcal mol^–1^) that offsets an unfavorable entropy (−*T*Δ*S* of 1.6 ± 1.6 kcal mol^–1^). A similar trend was observed for the binding of ADP and NAD^+^ to domain I (Table 1). The ligand-to-receptor ratio was substoichiometric (*n* = 0.6), as observed for other domain I interactions (Table 1). The binding of AP_2_-ribavirin to the full-length *Aai* riboswitch produced a slightly poorer *K*_D_ of 30.2 ± 1.2 μM, comparable to binding by NAD^+^ (Figure 5b and Table 1). The AP_2_-ribavirin enthalpic and entropic features mirrored those of NAD^+^. Notably, the ligand-to-receptor ratio was nearly 1.0, as observed for NAD^+^ (Table 1). The results suggest that a smaller uncharged triazole ring in place of the charged pyridine does not significantly alter cofactor recognition. Although NAD^+^ and AP_2_-ribavirin both exhibit pendant amide groups, the smaller size of the triazole could alter the hydrogen bond length and geometry at this position; this effect was not detected by our analysis. The binding of AP_2_-ribavirin is consistent with structure–activity relationship data in which amide or carboxylate groups on the pyridine ring (i.e., NAD^+^ or NaAD) are tolerated by domain I.^9^

### Evidence of Self-Association by Individual *nadA* Domains

**Figure 6:**
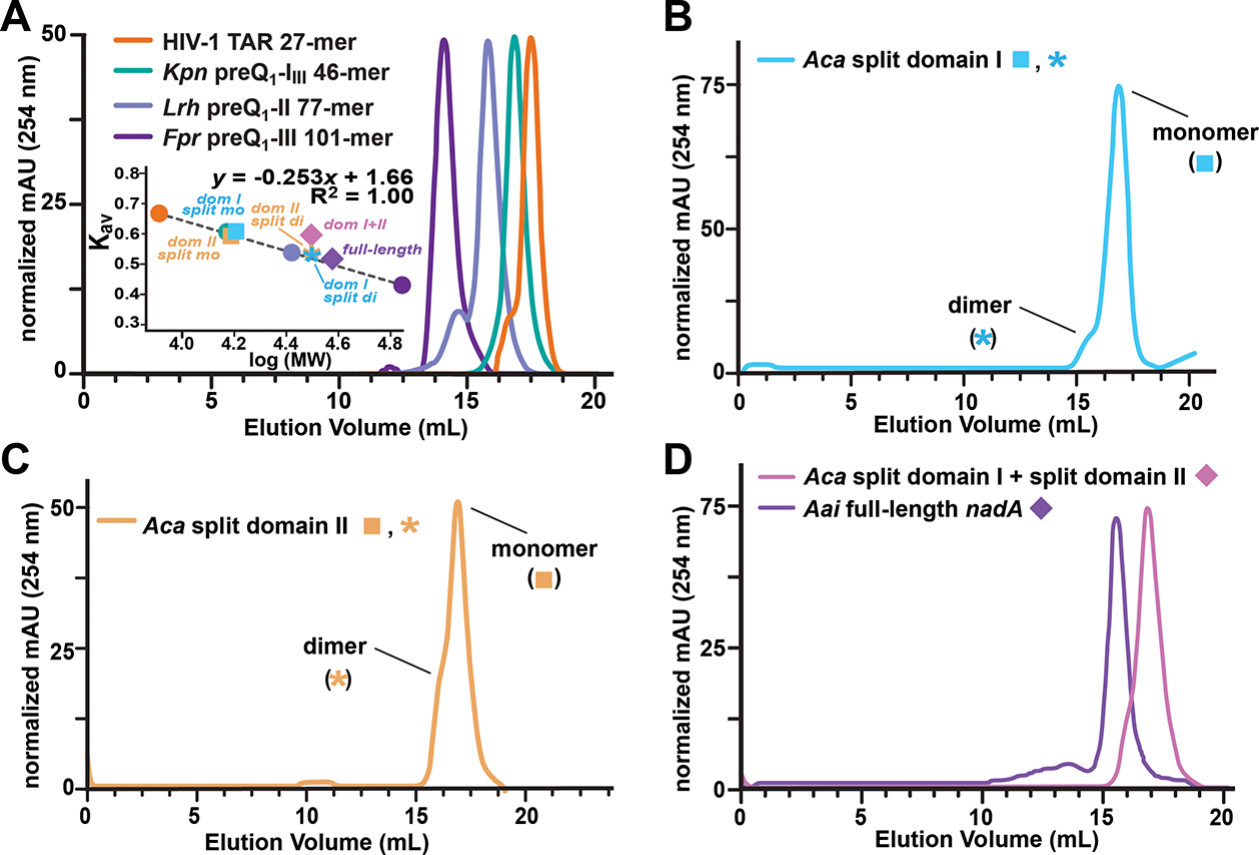
Representative size-exclusion chromatography (SEC) analysis of various *nadA* riboswitch constructs. (a) Molecular weight standards and calibration curve based on folded RNAs. Here and elsewhere the colored symbols correspond to the elution profiles provided; asterisks indicate the domain I and domain II split construct dimer peaks (i.e., prepeak shoulder maxima in elution profiles) mapped to the calibration curve and labeled “dom I split di” or “dom II split di”. The standards are indicated by colored circles and were normalized based on absorbance. (b) SEC profiles for the *Aca* domain I split constructs. (c) SEC profiles for the *Aca* domain II split constructs. (d) SEC profiles for the *Aca* domain I and domain II split forms (combined) and the full-length *Aai* riboswitch.

Oligomerization of individual *nadA* riboswitch domains has the potential to influence binding analysis by ITC. Accordingly, we analyzed each individual split construct domain and the full-length riboswitch by size-exclusion chromatography (SEC) in the presence of NAD^+^. As a control experiment, we calibrated our column by use of domains from well-folded RNAs characterized in our laboratory by SEC, ITC, and X-ray crystallography. These RNAs were chosen for calibration because they do not self-interact appreciably^37,38^ or they form predictable dimers at the concentrations used in chromatography (i.e., the *Fpr* preQ_1_-III riboswitch).^39^ The associated elution profiles and resulting standard curve are provided (Figure 6a). Importantly, a plot of *K*_av_ versus log(MW) obeys a linear relationship, as expected for compact biomacromolecules.^50^

SEC analysis of the *Aca* domain I split construct 50-mer revealed that the sample migrates mostly as a monomer (Figure 6b). This result is reasonable because the split construct comprises two strands that cannot form self-complementary dimers (Figure S1d). Indeed, the major peak of the domain I split construct migrates as a monomer that obeys the linear trend set by the RNA standards (Figure 6a, cyan square). Notably, a small shoulder is observed in the gel-filtration profile (Figure 6b), which runs as a dimer based on the standard curve (Figure 6a, cyan asterisk). This observation suggests that the split domain I construct self-associates through noncanonical interactions because it is unable to form self-complementary dimers. Indeed, any misfolded hairpin monomers of the split construct (i.e., 28-mers or 22-mers) would migrate slower than the monomer peak, whereas bimolecular homodimers would appear to run as monomers (i.e., 56-mers or 44-mers). By contrast, SEC analysis of domain I hairpin sequences yielded a bimodal elution profile dominated by dimers (Figure S5a,b). This result was likely caused by a mixture of dimers formed by complementary intermolecular WC pairing (Figure S1a,b) as well as the correctly folded intramolecular hairpin monomer (Figure S1c). This finding justifies our use of split domain constructs instead of a hairpin sequence for ITC analysis.

We next analyzed the *Aca* domain II split construct, which produced an asymmetric peak with a prominent prepeak shoulder (Figure 6c). The elution volume of the main peak was consistent with a monomer based on the calibration curve (Figure 6a, tan square), similar to split domain I (Figure 6a, cyan square). The prepeak shoulder eluted faster than the main peak and was consistent with a dimer based on the calibration curve (Figures 6a and c, tan asterisk). This finding suggests that the split domain II construct self-associates via noncanonical interactions because it is unable to form self-complementary dimers, as described above for split domain I. Like the hairpin sequence of domain I, the monomeric hairpin form of domain II was also accompanied by dimers and faster eluting species (Figures S5a and c). Again, these findings illustrate why the domain II hairpin sequence was unsuitable for our ITC analysis.

Finally, SEC analysis of the full-length *Aai nadA* riboswitch revealed that the riboswitch migrates as a compact structure in the presence or absence of NAD^+^, resulting in a single eluted peak (Figures 6d and S6); this suggests that the riboswitch is prefolded prior to effector binding. The *K*_av_ versus log(MW) analysis of the full-length riboswitch indicates that it obeys the linear trend established by the calibration curve (Figure 6a, purple diamond). By contrast, a comparable SEC analysis of combined domain I and domain II split constructs, added together after folding each individual domain, showed that the two domains do not associate with sufficient affinity to compose a single riboswitch in the presence of NAD^+^ (Figure 6a,d, pink diamond). Nonetheless, a prepeak shoulder in the split constructs elution profile suggests that some level of dimerization exists, albeit multiple intermolecular species are possible, including domain I–domain I, domain II–domain II, and domain I–domain II.

### Conserved Junction J1-1a and J1b-1a Nucleotides Make Key Long-Range Interactions

**Figure 7:**
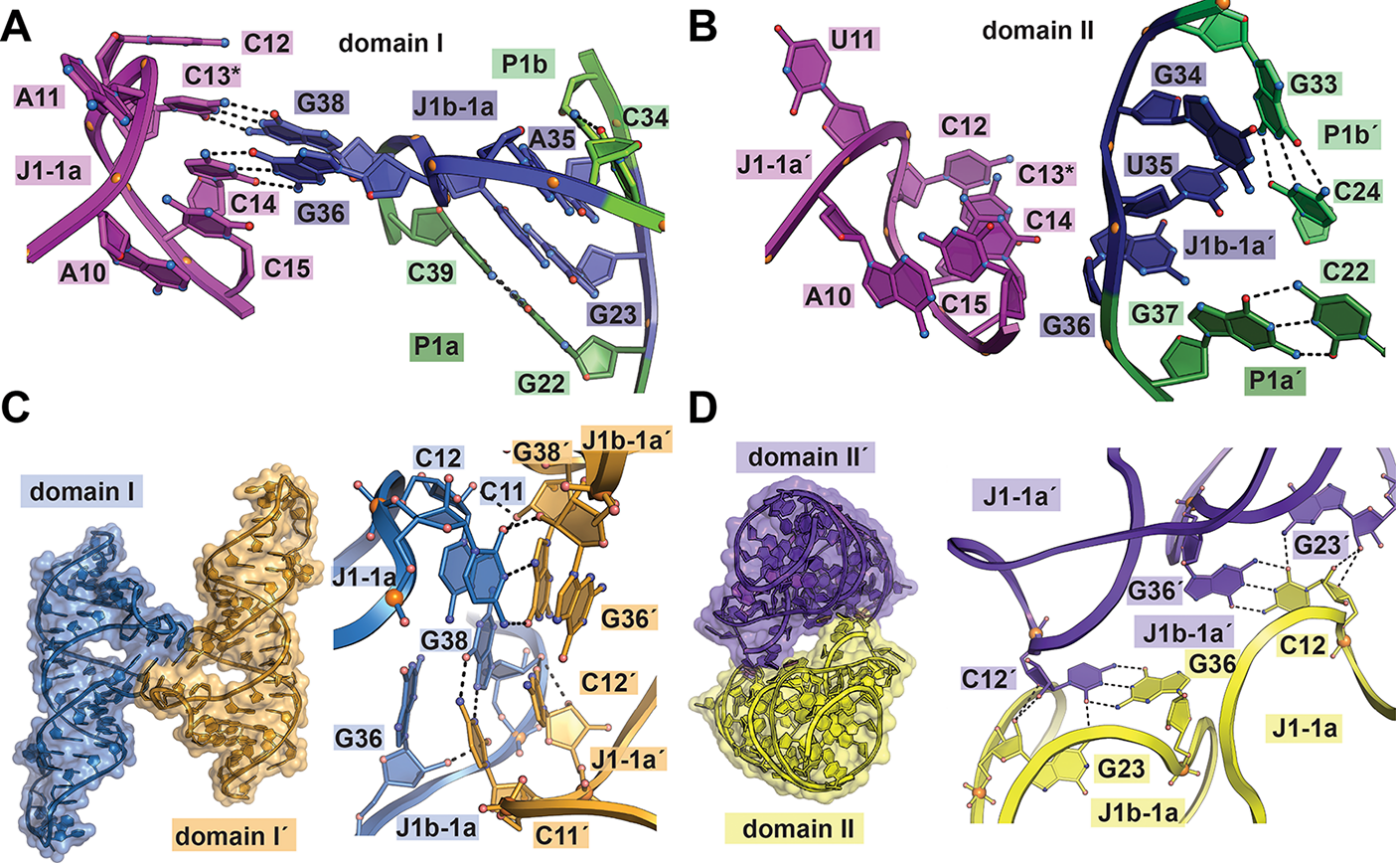
Intradomain contacts observed in the junctions of the domain I and II crystal structures and the interdomain crystal contacts in these regions. (a) Ribbon diagram of domain I (PDB 7d7w)^22^ showing intramolecular hydrogen bonds between conserved junction nucleotides in J1-1a and J1b-1a. C13 is noted by an asterisk (*) because of its importance in functional analysis.^9^ Putative hydrogen bonds are depicted as dashed lines. (b) Ribbon diagram of domain II (PDB 7d81)^22^ reveals no intramolecular hydrogen bonds between conserved junction nucleotides in J1-1a′ and J1b-1a′. C13 is noted by an asterisk (*) because of its importance in functional analysis.^9^ (c, left) Ribbon diagram of domain I interactions between crystallographically related molecules (PDB 7d7v(22)). (right) Close-up ball-and-stick view of the junction interface between J1-1a and J1b-1a in molecules related by crystallographic symmetry. (d, left) Ribbon diagram of domain II interactions between crystallographically related molecules (PDB 7d81(22)). (right) Close-up ball-and-stick view of the junction interface between J1-1a′ and J1b-1a′ in molecules related by crystallographic symmetry.

Breaker *et al*. hypothesized previously that domains I and II form an interface that promotes a single NAD^+^ binding pocket.^9^ A potential site for interdomain contacts is the nexus of the binding-pocket-proximal J1-1a junction and the distal J1b-1a junction (Figures 1d and 7a). Mutations in the domain I junction (e.g., C13G/G38C and C14G/G36C) impaired ligand binding by ablating intradomain contacts adjacent to the ligand binding pocket.^22^ Functional analysis of the full-length *nadA* riboswitch revealed that C13A impairs *nadA* riboswitch repression activity,^9^ consistent with disruption of the long-range C13/G38 interaction^22^ (Figure 7a).

By contrast, the structure of isolated domain II does not show intradomain J1-1a′/J1b-1a′ interactions^22^ (Figure 7b). This point is notable because the homologous C13A mutation in J1-1a′ of domain II produced a substantial loss in gene regulation in the context of the full-length *nadA* riboswitch, and the C13A double mutation (i.e., one mutant in each domain) produced a major loss in gene-regulatory function.^9^ Surprisingly, the crystal structure of isolated domain II suggests that C13 bulges into solvent^22^ (Figure 7b). Hence, the functional data^9^ and lack of intradomain interactions at C13 suggest that junction residues in domain II could play an important role in the fold and function of the full-length *nadA* riboswitch. This point is underscored by the 90–97% sequence conservation of nucleotides in this region.^9^

Although intramolecular interactions between domains I and II are not observable in the cocrystal structures of the isolated domains, we examined the crystallographic interfaces formed by each domain to see whether the J1-1a and J1b-1a sequences make interdomain contacts that could provide insight into the full-length structure. The occurrence of such contacts could also explain why each isolated split domain construct formed minor dimeric species in our SEC experiments, in contrast to the full-length riboswitch (Figures 6a–d).

Analysis of *Aai* domain I (PDB 7d7v)^22^ revealed a notable intermolecular interface involving J1-1a and J1b-1a. This interface contains eight hydrogen bonds derived from the bulged cytosines C11 and C12 in J1-1a and the bulged guanines G36′ and G38′ in J1b-1a′, which were donated from a symmetry-related molecule (Figure 7c). Specifically, the WC face of C11 hydrogen bonds to the Hoogsteen edge of G38′. In addition, the sugar edge of C12 hydrogen bonds to the 2′-OH group of G38′; the 2′-OH of G36′ also hydrogen bonds to the O2 keto of C11. A second *Aai* packing interface occurs between J1-1a and J1b-1a in a different domain I structure (PDB 7d7w),^22^ which reveals eight interdomain contacts (Figure S7a). Here, A11 of J1-1a uses its N6 exocyclic amine to interact with the sugar edge of C15′ of J1-1a′; the A11 base also stacks on the G36′ of J1b-1a′. A Mg^2+^ bridges the phosphate backbones of A11′ in J1-1a′ and U17 in J1-1a. A third unique intermolecular J1-1a and J1b-1a′ interface occurs in the *Ckv nadA* riboswitch (PDB 6tf1)^11^ (Figure S7b). Here, the bulged base A11 of J1-1a stacks with the G36′ of J1b-1a′ while simultaneously coordinating to Na^+^ (i.e., by A11 and C15′) and Mg^2+^ (i.e., by the phosphate backbone of A11 and U17′), which bridges the intermolecular interface. These observations imply that interactions between J1-1a and J1b-1a are influenced by mono- and divalent ions.

Domain II is homologous to domain I in terms of the consensus model.^9^ Although only a single crystal structure exists of domain II, the corresponding interface analysis (PDB 7d81)^22^ showed that J1-1a and J1b-1a engage in 12 intermolecular contacts, comparable to those observed for domain I. Notably, WC hydrogen bonds occur between C12/G36′, and the sugar edge of G23 in J1b-1a interacts with that of C12′ (Figure 7d). These interactions likely occur because domain II does not make long-range intradomain contacts between J1-1a cytosines and J1b-1a guanines (Figure 7b),^22^ thus allowing unpaired cytosines and guanines to participate in lattice interactions in the crystal structure. A take-home message is that each isolated domain exhibits multiple bulged bases in conserved junctions that can support long-range interactions. We posit that such contacts are important for intradomain contacts in the full-length *nadA* riboswitch.

Although we remain skeptical that the crystal packing interactions observed in the domain I and II crystal structures mimic the true *nadA* intramolecular interface, our analysis reveals the plausibility of interactions between the J1-1a and J1b-1a junctions. Such interactions could explain the propensity of the isolated domains to form homodimers in solution, as observed by SEC (Figures 6a–c). Formation of homodimers provides a rationale for the low *n* values observed in domain I ITC experiments (average *n* value of 0.68 ± 0.17, Table 1). For example, two domain I molecules could form an inactive dimer unreceptive to ligand binding or a dimer that binds only one ligand equivalent.

## Discussion

The goal of this work was to probe the ligand specificity and ligand-to-receptor ratios of the full-length *nadA* class I riboswitch. As a basis for comparison to previous studies, we investigated each domain in isolation as well as the full-length riboswitch, which was not examined in previous ITC and fluorescence studies.^11,22^ Understanding the basis of NAD^+^-sensing by regulatory RNAs provides insight not only into gene regulation by extant bacteria but also the capabilities of ancient RNAs that used NAD^+^ for redox reactions and metabolism in an RNA world.^21^ Our analysis of the full-length *nadA* riboswitch supports a model in which a single cofactor is recognized by a folded, globular riboswitch comprising domains I and domain II. A single-site ligand binding model produced the best fit to thermograms derived from titrations of ADP, NAD^+^, NADH, ATP, and AP_2_-ribavirin into the full-length riboswitch (Figures 4 and 5b). By contrast, two-site simulations produced poorer fits based on visual inspection (Figures S3c and d) and worse χ^2^ values. Notably, the average overall *n* value (i.e., ligand-to-receptor ratio) for the binding of a one-site ligand to the full-length riboswitch was 1.24 ± 0.21 (Table 1). This stoichiometry is consistent with an independent analysis of the full-length *nadA* motif, in which the ligand dependence of in-line probing was fit to the Hill equation, yielding a Hill coefficient of 1.0 (i.e., no cooperativity). Indeed, we observed no evidence of two-site binding using sequential or interdependent-sites binding models (Figures S3c and d). Notably, each binding model is expected to produce distinctive thermogram features, such as two inflections or parabolic curves characteristic of two-site binding,^45,51^ which were not observed in our experimental data. Interestingly, we observed that the entropic and enthalpic binding contributions for a particular ligand were similar for both domain I and the full-length riboswitch (Table 1). The similarity between entropic terms for the same ligand could be the result of comparable conformational or solvation changes upon ligand binding.^52^ Likewise, the closely related enthalpic components imply similar modes of ligand recognition by domain I and the full-length riboswitch. These observations have implications for the ligand binding model, which appears to be localized to a single domain that recognizes the ADP moiety (discussed below).

A major finding from previous studies was that domain I of the *nadA* riboswitch is necessary and sufficient for ADP recognition.^9,11,22^ Our work corroborates these observations and reveals that ADP, NAD^+^, NADH, and AP_2_-ribavirin each bind to *Aca* domain I with *K*_D_ values between 11.1 ± 0.6 and 55.5 ± 3.6 μM (Table 1). The affinity trend observed in terms of binding to the full-length *nadA* riboswitch was similar and followed the trend ADP = ATP > NAD^+^ = AP2-ribavirin > NADH. Cocrystal structures of domain I from *Ckv* and *Aca* show that the adenosyl moiety of NAD^+^ is recognized specifically in a binding pocket that comprises P1 and J1-1a (Figure 1d). Accordingly, the preponderance of evidence supports domain I as the major site of ADP recognition. Interestingly, the *nadA* riboswitch shows ∼30-fold higher affinity for ADP in our experiments compared to the *ykkC*-2c riboswitch, which specifically binds dNDP or NDP nucleotides.^53^

In contrast to domain I, domain II of the *nadA* riboswitch showed no detectable binding to NAD^+^ or other analogues under the conditions of our ITC analysis (Figure 3 and Table 1). Our findings corroborate previous in-line probing analyses in which ten domain II variants from different organisms were tested for binding to NAD^+^ and other analogues.^9^ Ligand-dependent modulation of the structure was not detected in these constructs nor in those with tandemly arranged domains I and II.^9^ However, the binding of domain II to ADP was detected using sensitive 2-aminopurine fluorescence measurements using RNA derived from *Acidobacteriaceae* bacterium KBS 83 in a buffer containing 50 mM Mg^2+^. Data fit to a single-site binding model produced an apparent *K*_D_ of 3.4 ± 0.5 mM.^22^ Although weak, this finding appears to support NAD^+^ binding observed in a domain II crystal structure, which occurred at a dyad interface produced by crystal packing.^22^ Accordingly, it was hypothesized that domain II possesses a cryptic NAD^+^ binding site in the context of the full-length riboswitch.^22^ However, our analysis of domain II and the full-length riboswitch here, as well as ITC simulations of the full-length *nadA* riboswitch (Figures S3c and d), cannot confirm this proposition.

In terms of the *nadA* gene regulatory mechanism, we note that an NAD^+^*K*_D_ of 3.4 mM for domain II (assuming ADP binds with the same affinity as NAD^+^) would necessitate an effector concentration ∼10-fold higher than its *K*_D_ to produce saturation of the domain II binding pocket. Accordingly, cells would need to reach an astonishingly high concentration of ∼34 mM NAD^+^ to achieve full binding and gene regulation, i.e., a level 13-fold higher than the 2.6 mM concentration of NAD^+^ measured in aerobically growing, glucose-fed *E. coli*.^23^ Remarkably, *E. coli* cells that were fed NAD^+^ for 26 h reached cofactor levels of only 6.2 mM.^54^ Accordingly, we posit that the binding of NAD^+^ to domain II in the crystal structure is an idiosyncrasy of its propensity to self-associate, which may have resulted in the weak apparent *K*_D_ of 3.4 mM measured outside the context of the full-length riboswitch.

The main difficulties in dissecting the functional relevance of individual *nadA* riboswitch domains include their high degree of sequence homology^9^ and their ability to self-interact (Figures 6a–c). Indeed, functional *nadA* riboswitches require both domains for gene regulation in bacteria, and no known examples exist of single-domain *nadA* riboswitches.^9^ Our SEC results showed that isolated domains I and II are prone to self-interaction outside the context of the full-length riboswitch (Figures 6a–c). Although the basis for these intermolecular contacts is unknown, natural intradomain long-range interactions and crystal packing show that the helical junctions (i.e., J1-1a and J1b-1a) are “sticky” due to conserved cytosine and guanine tracts (Figures7 and S7). As such, the “sticky” properties of isolated domains are likely an underlying factor leading to the reduced ligand-to-receptor ratios (*n* values) observed by ITC for domain I (Table 1). Specifically, each ligand shows substoichiometric *n* values ranging from 0.5 for ADP to 0.9 for NADH. The average *n* value for all domain I binding experiments was 0.68 ± 0.17. This value suggests that up to one-half of domain I molecules were incompetent for binding ligands. Such a scenario would arise if domain I molecules formed intermolecular dimers during ITC, leading to the formation of a pseudoriboswitch competent enough to form one ligand binding pocket. Notably, substoichiometric *n* values were reported independently for *Aca* riboswitch domain I binding to ADP (*n* = 0.83 ± 0.06).^22^ Hence, only the full-length riboswitch appears to be capable of producing a competent ligand binding pocket, as indicated by an average ligand-to-receptor ratio of 1.2 ± 0.2 based on Table 1.

Our inability to reconstitute an intact riboswitch from individual domain I and domain II constructs (Figure 6d) was somewhat unexpected, given precedents in the field. For example, a structured, active hairpin ribozyme could be formed by mixing individually folded loop A and loop B domains.^55,56^ Independently folded aptamers of the tandem glycine riboswitch interacted in a *trans*, glycine-dependent manner.^57^ At present, it is unclear why domains I and II of the *nadA* riboswitch did not form a complex, although removal of the interdomain linker could have eliminated a critical structural element required for complex formation. Interdomain contacts between aptamer 1 and aptamer 2 of the glycine riboswitch utilize multiple patches of A-minor contacts between P1 helices and junction or loop regions that form reciprocal contacts (Figure S9a).^58^ The hairpin ribozyme uses interdomain WC pairing at a sharp S-turn bend and a nearby ribose zipper motif to support the docking of loop A and loop B (Figures S9b and c).^55^ Although the *nadA* sequence does not exhibit extended adenine sequences suggestive of A-minor interactions, single conserved adenines are present in the J1-1a consensus model that could be employed for tertiary interactions. It is also plausible that one or more WC or ribose zipper interactions occur between domains, originating from J1-1a and J1b-1a.

**Figure 8:**
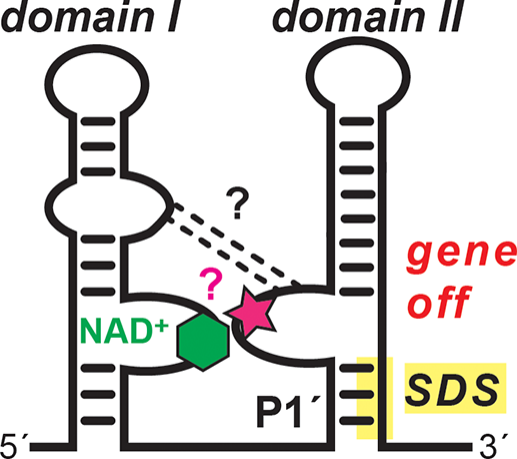
Model for gene regulation by the *nadA* riboswitch. Evidence suggests that NAD^+^ (hexagon) is recognized by domain I via its ADP moiety. In our model, domains I and domain II are hypothesized to dock using interactions (dashed lines) between conserved bases in junction regions, possibly mediated by J1-1a and J1b-1a. The bound state of domain I assists domain II folding, which sequesters the Shine-Dalgarno sequence (SDS) in P1′ and leads to a gene-off state. The presence of auxiliary factors (e.g., ions, small molecules, or proteins, depicted as a pink star) that impart NAD^+^ specificity or intradomain docking cannot be ruled out.

Intriguingly, structural and functional parallels exist between the *nadA* riboswitch and the tandem glycine riboswitch wherein the latter also comprises two homologous but nonidentical aptamers connected by a linker.^59,60^ Although each glycine riboswitch aptamer binds a single ligand, glycine binding to aptamer 1 was more sensitive to intramolecular dimerization than aptamer 2.^57^ Moreover, aptamer 2 showed much weaker affinity for glycine. The posited gene regulatory model suggests that ligand binding to aptamer 1 is linked to dimerization and stabilization of P1 within aptamer 2, which controls the expression platform.^58^ Accordingly, domain I of the *nadA* riboswitch is primarily responsible for NAD^+^ recognition, which ostensibly provides a scaffold that promotes domain II folding. Like the glycine riboswitch, domain II contains the expression platform, which is likely buried upon formation of the P1′ helix, leading to a gene-off state (Figure 8).

One of the most unexpected findings was that the full-length *nadA* riboswitch binds ATP and that its affinity is comparable to ADP (Figures 4a and d and Table 1). This result was surprising because most riboswitches show high specificity for their effectors,^1^ although exceptions such as the azaaromatic riboswitch, which senses multiple effectors, exist.^61^ Cobalamin riboswitches also exhibit a broad range of ligand discrimination abilities,^62^ with some atypical variants capable of sensing multiple ligands based on intrinsic structural adaptability.^63^ The *ykkC*-2c riboswitch recognizes dNDPs and NDPs, suggesting it does not select specific nucleobases or sugars based on a 2′-hydroxyl group.^53^ However, the *ykkC-*2c riboswitch does discriminate against the γ-phosphate of NTPs, which do not bind as tightly as nucleoside diphosphate.^53^ Although the NAD^+^-II (class II) riboswitch also binds NAD^+^, it does not recognize the cofactor’s adenosyl moiety, in contrast to the *nadA* (NAD^+^-I) riboswitch herein. This is because the NAD^+^-II aptamer senses the nicotinamide riboside moiety of the cofactor,^64−66^ which also results in affinity for NMN and NR because they are substructures of NAD^+^. Thus, despite the hypothesis that *nadA* domain II would bind the nicotinamide moiety of NAD^+^ in the context of the full-length riboswitch,^9^ we did not observe this, and the γ-phosphate of ATP is accommodated readily (Figure 4d and Table 1). In mid-log phase Gram-positive *E. coli*, ATP levels are 9.6 mM, whereas NAD^+^ is 2.6 mM.^23^ In Gram-negative *Clostridium autoethanogenum* undergoing acetogenesis and solventogenesis, ATP and NAD^+^ concentrations were measured at concentrations of 118–134 μM and 1.6–1.7 mM, respectively.^24^ These ATP and NAD^+^ concentrations suggest that both effectors will saturate the *nadA* riboswitch binding site based on the *K*_D_ values of 11.0 ± 3.5 μM for ATP and 31.5 ± 1.5 μM for NAD^+^ measured here (Table 1). Lack of strong binding specificity by the *nadA* riboswitch and others, such as the azaaromatic, cobalamin, and *ykkC-*2C riboswitches noted above, could signify that lax ligand preference is more common than realized previously. On the other hand, the *nadA* motif associates exclusively with NAD^+^ biosynthesis genes, but not those involved in ATP regulation.^8^ Whether the *nadA* riboswitch is truly promiscuous in ligand binding or represents a less evolved riboswitch compared to its NAD^+^-II counterpart remains to be seen. The latter possibly would be surprising because riboswitch function is known to promote organismal fitness *in vivo*.^67^

Little is known at present about the regulation of bacterial NAD^+^ biosynthesis,^68^ although the NadE protein (Figure 1c) is known to interact with the regulatory transducing protein PII, which simultaneously senses ATP, ADP, α-KG, and l-Gln,^69^ i.e., essential metabolites that report on the cellular nutrient state.^70^ In this system, ATP and ADP compete for PII binding such that high α-KG levels cooperatively enhance Mg–ATP affinity,^71^ ablating the NadE–PII interaction.^68^ The possibility that additional ligands, proteins, or ions could participate in *nadA* riboswitch sensing and function remains a possibility.^9^ For example, K^+^ ions increase the affinity of the lysine riboswitch for its amino acid effector through synergistic binding.^47,48^ Although added K^+^ ions had no effect on NAD^+^ affinity here (Figure S8), other unidentified factors could be required to impart the level of specificity needed for the *nadA* riboswitch to exclusively sense NAD^+^ (Figure 8). The identification of such factors remains a challenge.

## Supporting Information Available

Predicted secondary structures of domain I and domain II (Figure S1), electrospray ionization mass spectrometry analysis of NAD^+^, ADP, NADH, AP_2_-ribavirin, and ATP (Figure S2), ITC simulations under low affinity conditions and tests of various two-site binding models (Figure S3), representative ITC injection and titrations of ligands into an ITC buffer (Figure S4), size-exclusion chromatography of the hairpin domain I and hairpin domain II (Figure S5), size-exclusion chromatography of the full-length *Aai* riboswitch in the absence of NAD^+^ (Figure S6), interdomain junction interfaces of *nadA* riboswitch domain I (Figure S7), representative ITC thermogram of ligand titration into the full-length *Aai* riboswitch in Na^+^ and K^+^ (Figure S8), and intradomain interactions found in two-domain riboswitches and ribozymes (Figure S9) (PDF)

## Abbreviations

NAD^+^: nicotinamide adenine dinucleotide
NADH: nicotinamide adenine dinucleotide hydrogen (reduced form)
ADP: adenosine diphosphate
AP_2_-ribavirin: 1,2,4-triazole-3-carboxamide adenine dinucleotide
ATP: adenosine triphosphate
*Aai*: *Acidobacterium ailaaui*
*Aca*: *Acidobacterium capsulatum*
WC: Watson–Crick
SEC: size-exclusion chromatography
ITC: isothermal titration calorimetry
*Ckv*: *Candidatus koribacter versatilis*
*Eag*: *Edaphobacter aggregans*
NaAD: nicotinic acid adenine dinucleotide
NMN: nicotinamide mononucleotide
NR: nicotinamide riboside
α-KG: α-ketoglutarate
l-Gln: l-glutamine
