# Supplementary material for: Full-Length NAD+-I Riboswitches Bind a Single Cofactor but Cannot Discriminate against Adenosine Triphosphate
Source: Biochemistry. 2023 Nov 10;62(23):3396–410. doi: 10.1021/acs.biochem.3c00391 (PMC10702441; doi:10.1021/acs.biochem.3c00391)
Supplement: Supplementary file 1 — bi3c00391_si_001.pdf [file bi3c00391_si_001.pdf]

Supporting Information

Full-length NAD^+-I riboswitches bind a single co-factor but cannot discriminate against adenosine triphosphate

Yoshita Srivastava[‡], Maya E. Blau[‡,†], Jermaine L. Jenkins[‡] and Joseph E. Wedekind[‡,]*

[‡]Department of Biochemistry & Biophysics and Center for RNA Biology, University of Rochester School of Medicine & Dentistry, Rochester NY 14642, USA

[†]Present address: Medical College of Wisconsin, Milwaukee, WI 53226

*Correspondence to: joseph.wedekind@rochester.edu

KEYWORDS: riboswitch, *NadA*, gene regulation, isothermal titration calorimetry, RNA structure, NAD^+, crystal structure, adenosine diphosphate, molecular recognition.

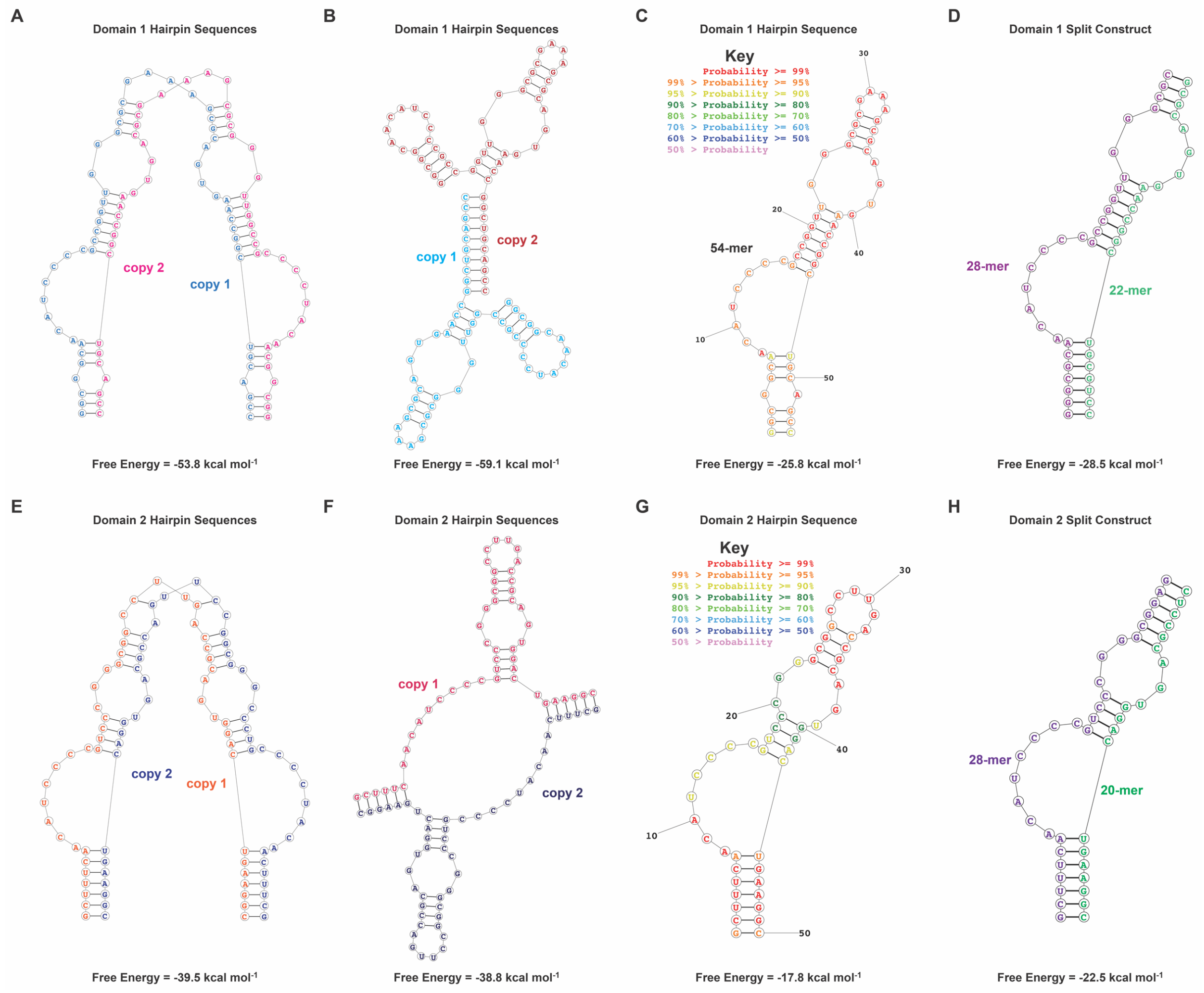

Figure S1. NAD^{+}-I riboswitch secondary structure predictions based on calculations using RNAstructure. (**A**) Lowest energy structure for the domain I 54-mer predicted by the Duplex algorithm reveals a dimer formed by intermolecular WC pairing (ΔG = -53.8 kcal mol^{-1}). (**B**) Lowest energy structure for the domain I 54-mer predicted by the Bifold algorithm reveals a dimer formed by intra- and intermolecular WC pairing (ΔG = -59.1 kcal mol^{-1}). (**C**) Lowest energy structure for the domain I 54-mer based on "predict secondary structure" reveals a hairpin formed by intramolecular WC pairing (ΔG = -25.8 kcal mol^{-1}). The hairpin is less stable than intermolecular dimers in **A** & **B**. (**D**) Lowest free energy structure for a split domain I construct comprising 28-mer and 22-mer strands. The results were identical using Duplex and BiFold. The intermolecular WC pairing produced a ΔG of -28.5 kcal mol^{-1}. Misfolding into intramolecular hairpins by the individual 28-mer and 22-mer was relatively weak, yielding ΔG values of -10.7 kcal mol^{-1} and -10.3 kcal mol^{-1}. (**E**) Lowest energy structure for the domain II 50-mer predicted as described in **A** (ΔG = -39.5 kcal mol^{-1}). (**F**) Lowest energy structure for the domain I 50-mer predicted as described in **B** (ΔG = -38.8 kcal mol^{-1}). (**G**) Lowest energy structure for the domain II 50-mer as described in **C** (ΔG = -17.8 kcal mol^{-1}). The hairpin is predicted to be less stable than intermolecular dimers in **E** & **F**. (**H**) Lowest free energy structure for a split domain II construct comprising 28-mer and 20-mer strands. The results were identical using Duplex and BiFold. The intermolecular WC pairing produced a ΔG of -22.5 kcal mol^{-1}. Misfolding into intramolecular hairpins by the individual 28-mer and 20-mer was very weak, yielding ΔG values of -5.8 kcal mol^{-1} and -1.8 kcal mol^{-1}.

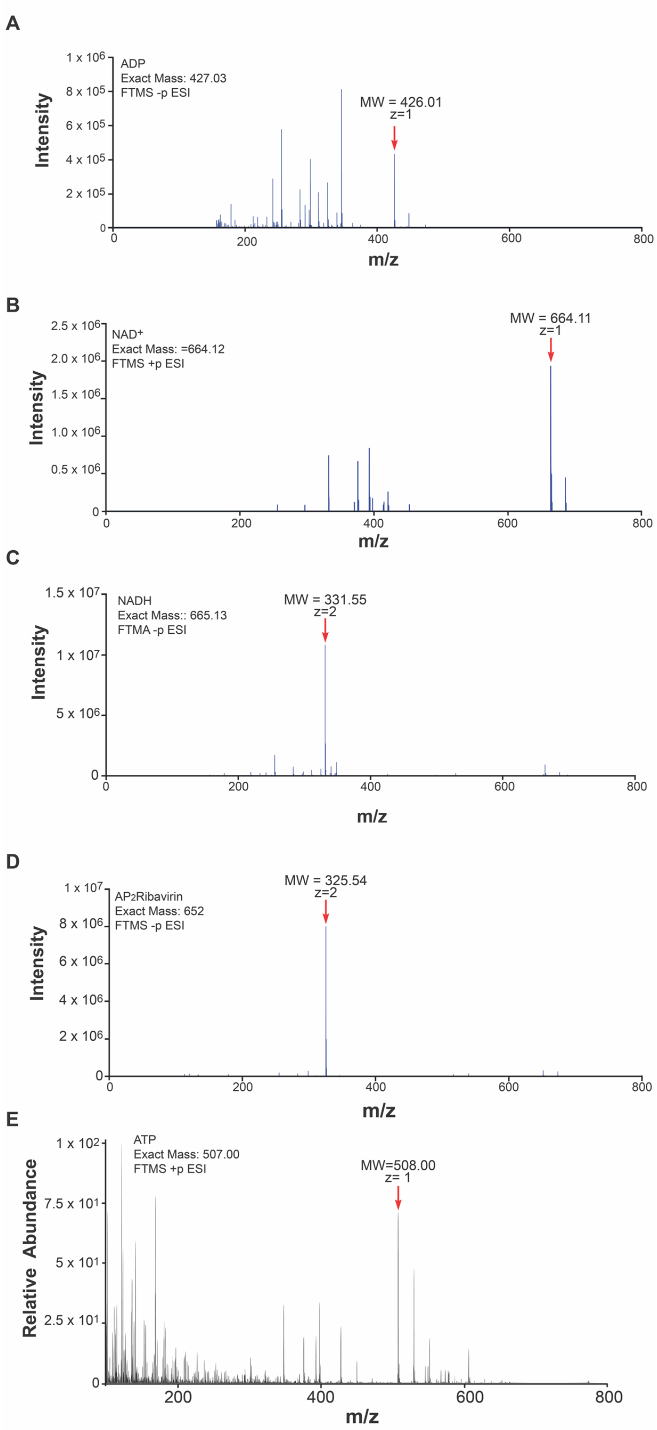

Figure S2. Electrospray ionization mass spectrometry analysis of NAD^+, ADP, NADH, AP_2Ribavirin and ATP. (**A**) ADP showed a peak at 426.01 corresponding to the expected m/z for ADP in negative ion mode. (**B**) NAD^+ showed a peak at 664.11 corresponding to the expected m/z for ADP in positive ion mode. (**C**) NADH showed a peak at m/z= 331.55 corresponding to the expected doubly charged species in negative ion mode. (**D**) AP_2-ribavirin showed a peak at m/z 325.54 corresponding to the expected doubly charged species in negative ion mode. (**E**) ATP showed a peak at m/z = 508.00 corresponding to the protonated species in positive ion mode.

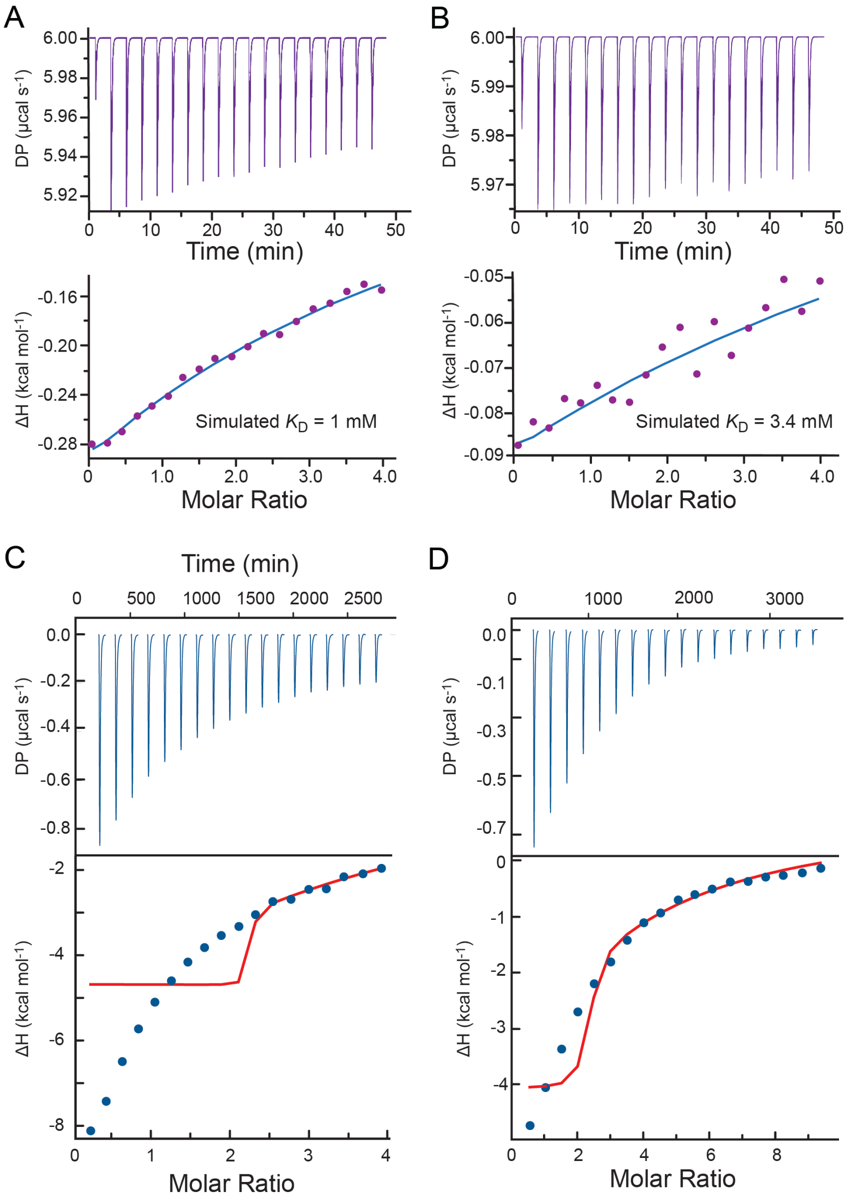

Figure S3. ITC simulations under low affinity conditions and tests of various two-site binding models. (**A**) Simulation using MicroCal PEAQITC Analysis Software indicating the ability to detect 1 site binding by domain II, although accurate fitting would not be possible. We assumed a K_D value of 1 mM with 1 mM ligand in the syringe and 50 µM riboswitch in the cell. The reference power was 6 µcal s^{-1}; the total heat was 7.6 µcal; feedback was high. (**B**) Simulation of domain II binding using conditions described in panel A, except that the K_D was set to 3.4 mM. (**C**) The simulated sequential-site binding model (solid line) for the full-length riboswitch showing the fit for a cell concentration of 30 µM and a syringe concentration of 630 µM, which was used to match experimental conditions (**Supporting Figure S3a**). The simulation assumes two high-affinity sites (K_{D1} of 30 µM and K_{D2} of 50 µM). Simulations were conducted using SEDPHAT. Experimental inputs were from NAD^+ titration into the full-length *Aai nadA* riboswitch (upper thermogram, closed circles). (**D**) The simulated sequential-site binding model derived using conditions described in panel C. The simulation assumes one high affinity site (K_{D1} of 20 µM) and one low affinity site (K_{D2} of 3.4 mM).

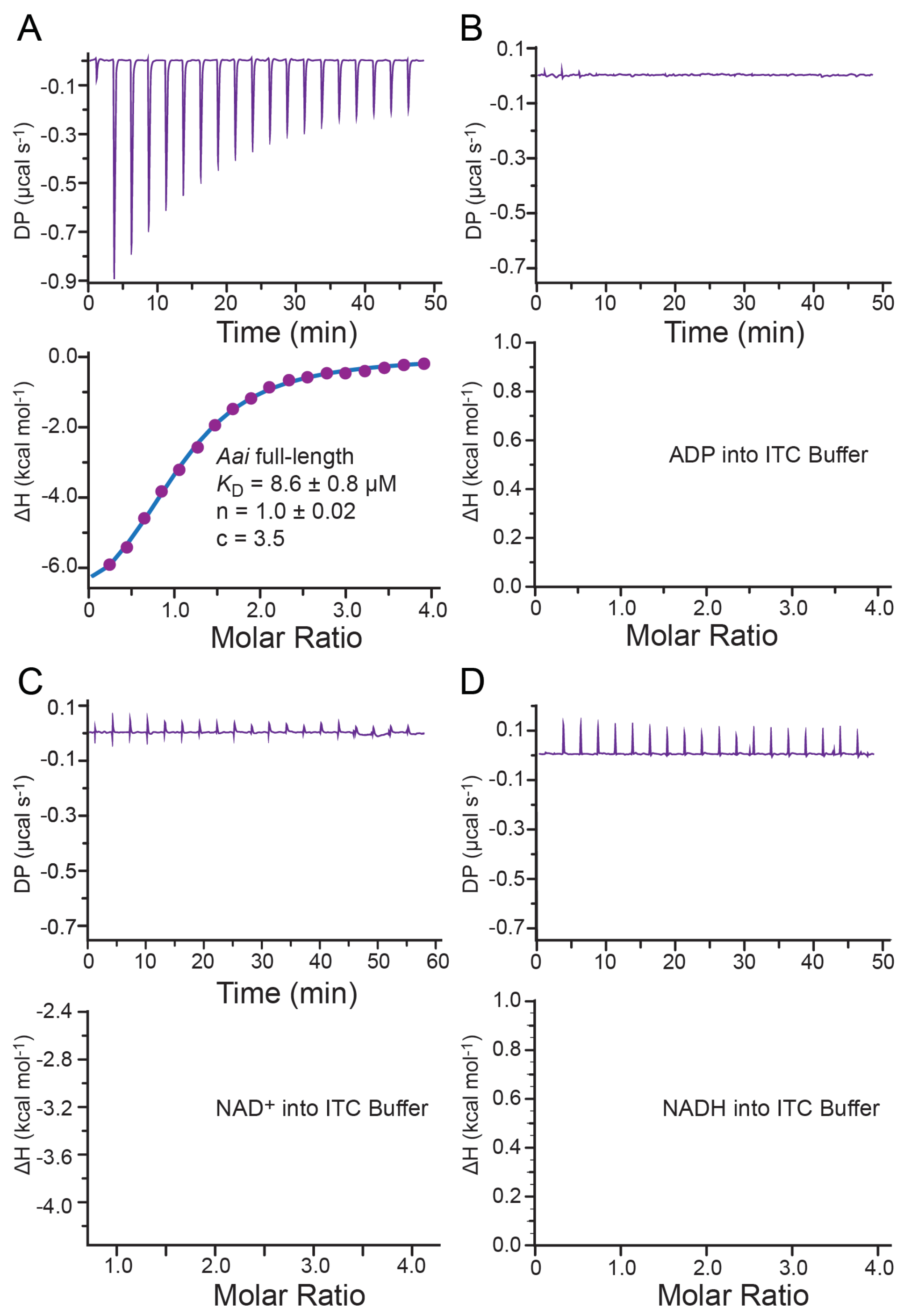

Figure S4. Representative ITC injection and titrations of ligand into ITC buffer. (**A**) Replicate thermogram and isotherm fit for ADP injection into the full-length *Aai nadA* riboswitch. (**B**). ADP in the syringe was injected into ITC buffer to measure the heat of dilution. The ligand concentration matched that of experiments reported in the main text. (**C**). NAD^+ in the syringe was injected into ITC buffer as described in panel B. (**D**) NADH in the syringe was injected into ITC buffer as described in panel B.

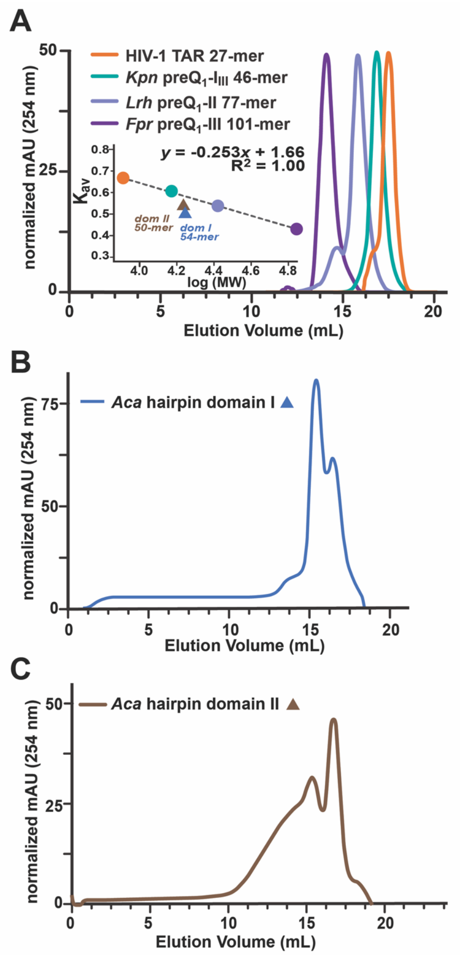

Figure S5. **Representative size-exclusion chromatography (SEC) analysis of various *nadA* riboswitch hairpin constructs.** (**A**) Molecular weight standards and calibration curve based on folded RNAs. Here and elsewhere the colored symbols correspond to elution profiles provided. The standards are indicated by colored circles and were normalized based on absorbance. (**B**) SEC profiles for the *Aca* domain I hairpin. Comparison of the first-peak elution volume (blue triangle) to the linear trend set by calibration standards shows that the sample does not fall on the curve as a monomer. (**C**) SEC profiles for the *Aca* domain II hairpin. Comparison of the first-peak elution volume (brown triangle) to the linear trend set by calibration standards shows that the sample does not fall on the curve as a monomer.

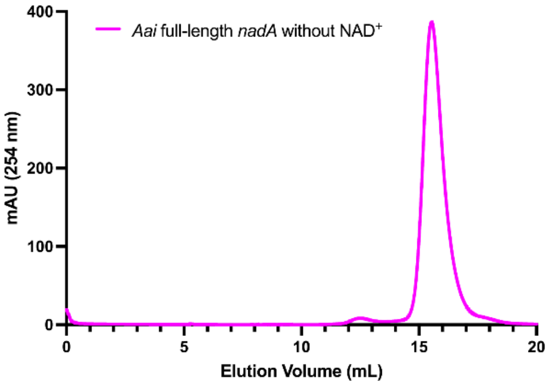

Figure S6. Size-exclusion chromatography of the full-length *Aai* riboswitch in the absence of NAD^+.

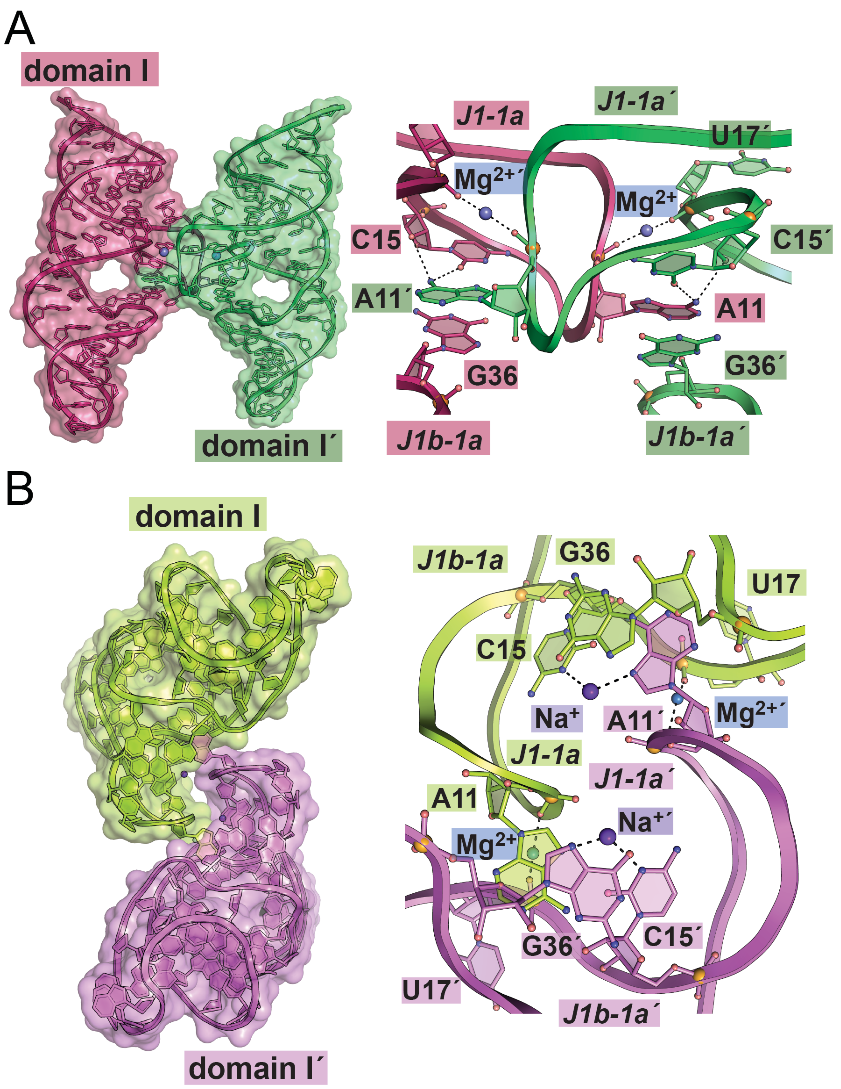

Figure S7. Interdomain junction interfaces of *nadA* riboswitch domain I. (**A**) A distinct interface in the *Aai* riboswitch (PDB entry 7d7w). Here and elsewhere, crystallographically related subunits are labeled domain I (red) and domain I´ (green). (**B**) Another distinct interface from the *Ckv nadA* riboswitch (PDB entry 6tf1).

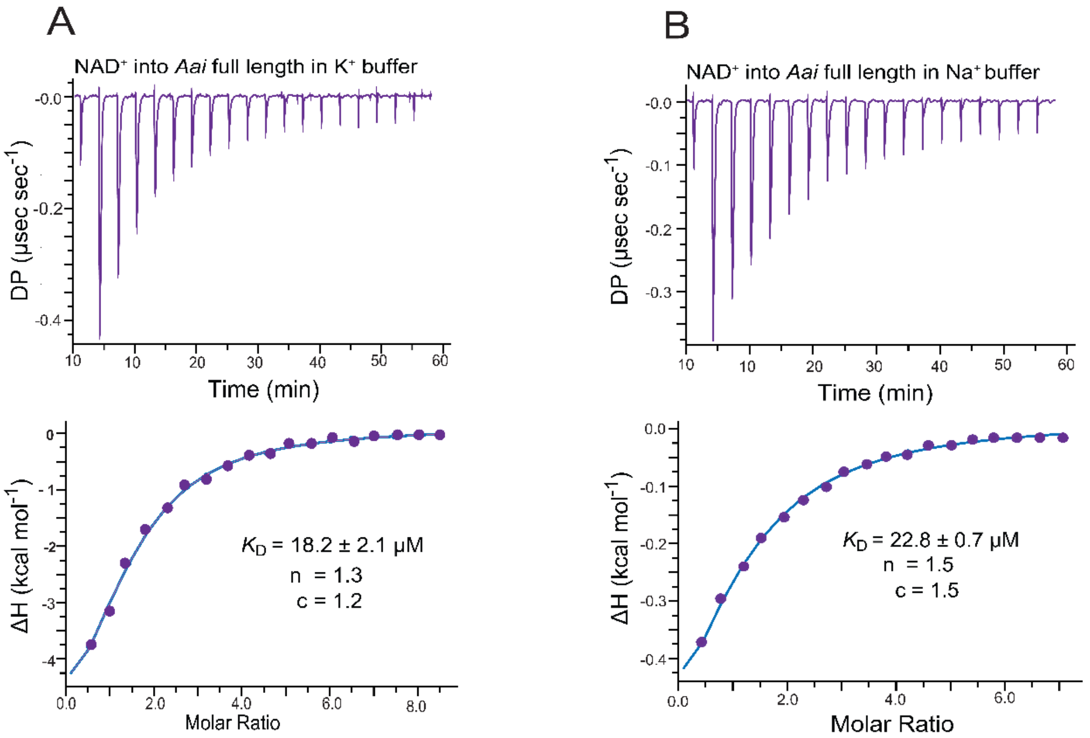

Figure S8. Representative ITC thermogram of ligand titration into the full-length *Aai* riboswitch in K^+ and Na^+ containing buffers. (**A**) ITC thermogram and isotherm fit for NAD^+ titration into the full-length *Aai nadA* riboswitch in KCl-containing ITC buffer. (**B**) Replicate thermogram and isotherm fit for NAD^+ titration into the full-length *Aai nadA* riboswitch in presence of NaCl-containing ITC buffer.

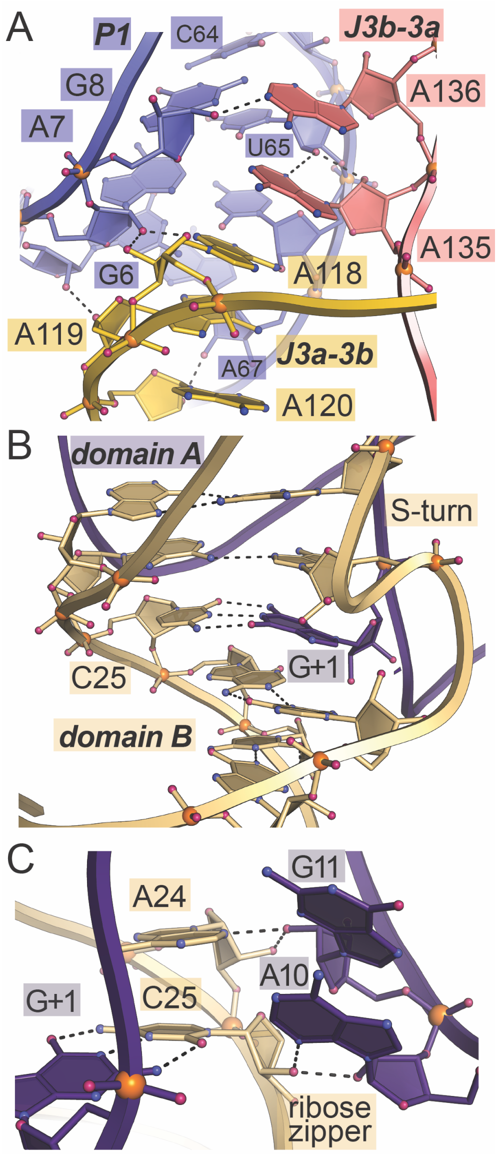

Figure S9. Intradomain interactions found in two-domain riboswitches and ribozymes. (**A**) Representative interaptamer interface of the tandem glycine riboswitch from *Fusobacterium nucleatum* (PDB entry 3p49). Inclined A-minor adenines from J3a-3b and J3b-3a (aptamer2) interact with helix P1 (aptamer1). Interactions include: A136, which forms a type I-like interaction with the G8-C64 pair; A135, which forms a type II interaction with the A7-U65 pair; A118, which forms a type II interaction with the A7-U65 pair; A119, which forms a type III-like interaction with G6-C66; and A120, which forms a type 0-like interaction with U4-A67 Here and elsewhere, putative hydrogen bonds are depicted as dashed lines. (**B**) Interdomain interface of the hairpin ribozyme (PDB entry 2oue). A canonical G+1-C25 base pair connects the two domains at the site of the S-turn, which extrudes nearby bases to make room for the cross-domain interaction. (**C**) A ribose zipper motif supports interdomain contacts between the loop A and B domain of the hairpin ribozyme. The view is rotated ~90° about the y-axis from panel **B**.
